# Dynamic gating-enhanced deep learning model with multi-source remote sensing synergy for optimizing wheat yield estimation

**DOI:** 10.3389/fpls.2025.1640806

**Published:** 2025-07-21

**Authors:** Jian Li, Junrui Kang, Jian Lu, Hongkun Fu, Zheng Li, Baoqi Liu, Xinglei Lin, Jiawei Zhao, Hengxu Guan, He Liu, Zhihan Liu

**Affiliations:** ^1^ College of Information Technology, Jilin Agricultural University, Changchun, China; ^2^ Jilin Province Cross-Regional Collaborative Innovation Center for Agricultural Intelligent Equipment, Jilin Agricultural University, Changchun, China; ^3^ College of Resources and Environment, Jilin Agricultural University, Changchun, Jilin, China; ^4^ College of Agriculture, Jilin Agricultural University, Changchun, China; ^5^ Northeast Institute of Geography and Agroecology, Chinese Academy of Sciences, Changchun, China; ^6^ College of Engineering and Technology, Jilin Agricultural University, Changchun, Jilin, China

**Keywords:** multi-source remote sensing, deep learning, wheat yield estimation, transformer, MoE module

## Abstract

**Introduction:**

Accurate wheat yield estimation is crucial for efficient crop management. This study introduces the Spatio–Temporal Fusion Mixture of Experts (STF-MoE) model, an innovative deep learning framework built upon an LSTM-Transformer architecture.

**Methods:**

The STF-MoE model incorporates a heterogeneous Mixture of Experts (MoE) mechanism with an adaptive gating network. This design dynamically processes fused multi-source remote sensing features (e.g., near-infrared vegetation reflectance, NIRv; fraction of photosynthetically active radiation absorption, Fpar) and environmental variables (e.g., relative humidity, digital elevation model) across multiple expert networks. The model was applied to estimate wheat yield in six major Chinese provinces.

**Results:**

The STF-MoE model demonstrated exceptional accuracy in the most recent estimation year (R² = 0.827, RMSE = 547.7 kg/ha) and exhibited robust performance across historical years and extreme climatic events, outperforming baseline models. Relative humidity and digital elevation model were identified as the most critical yield-influencing factors. Furthermore, the model accurately estimated yield 1-2 months before harvest by identifying key phenological stages (March to June).

**Discussion:**

STF-MoE effectively handles multi-source spatiotemporal complexity via its dynamic gating and expert specialization. While underestimation persists in extreme-yield regions, the model provides a scalable solution for pre-harvest yield estimation. Future work will optimize computational efficiency and integrate higher-resolution data.

## 1 Introduction

Wheat, as a cornerstone crop in the global agricultural system, yields approximately 800 million metric tons annually, supporting the dietary energy needs of over 35% of the global population. In China, wheat accounts for 92.3% of total summer grain production (2024), rendering its accurate yield estimation critical for agricultural policymaking, food security assurance, and international trade equilibrium (Chen et al, 2024).

Over the past decades, agricultural researchers have been dedicated to developing more precise crop yield estimation methods. Due to the complexity and limited applicability of conventional manual yield measurement techniques, remote sensing technology has achieved significant progress in agricultural monitoring by virtue of its rapid, cost-effective, and scalable advantages (Karthikeyan et al., 2020; Raza et al, 2025). Early remote sensing-based yield assessment primarily relies on vegetation indices and related parameters, which are derived from satellite remote sensing data to reflect crop growth status and health conditions. To further enhance estimation accuracy and mechanistic interpretability, advanced approaches such as light use efficiency (LUE)-based models and data assimilation have been extensively researched and applied (Lu et al., 2024a). The former quantifies crop yield potential by measuring the efficiency of light energy capture and conversion, while the latter integrates multi-source remote sensing observations into crop growth models to iteratively optimize simulation processes and state variables. Within these estimation frameworks—whether empirical-statistical or process-based—leaf area index (LAI) and fraction of photosynthetically active radiation absorption (Fpar) are recognized as core biophysical variables for yield estimation (Smith et al., 2019; Liang and Wang (2020). By quantifying canopy light interception efficiency, these parameters directly indicate dry matter accumulation dynamics and constitute fundamental descriptors of vegetation photosynthetic activity and canopy structure (Smith et al., 2019; Liang and Wang (2020). While these biophysical parameters offer direct insights, their estimation from satellite imagery often relies on vegetation indices. A widely used example, the traditional normalized difference vegetation index (NDVI), however, encounters issues such as spectral saturation under high biomass conditions. To address the spectral saturation limitation of the traditional normalized difference vegetation index (NDVI) under high biomass conditions, the green normalized difference vegetation index (GNDVI) was developed by replacing the red band with a green band, significantly enhancing chlorophyll sensitivity (Tian et al, 2020). Similarly, the enhanced vegetation index (EVI) introduces a blue band correction mechanism to effectively suppress soil background noise (Burns et al., 2022). Notably, the near-infrared reflectance vegetation index (NIRv) achieves accurate inversion of vegetation-absorbed photosynthetically active radiation through the multiplicative integration of NDVI and near-infrared reflectance (Mallick et al., 2024). In terms of environmental stress responses, embedding factors like solar radiation (Rad), soil organic carbon (SoC), and topography (DEM, Slope) into yield estimation models is essential. These parameters synergistically govern the crop’s microenvironment and physiological responses; for instance, Rad is the primary energy source, SoC influences nutrient-water availability, and topography dictates local environmental variations. Consequently, there is a growing scholarly focus on integrating these environmental and topographical variables with the previously discussed remote sensing-derived vegetation characteristics (e.g., various VIs, LAI, Fpar). Numerous studies suggest that such a comprehensive approach to parameter selection and combination can effectively enhance yield estimation accuracy and robustness (Fu et al., 2025a; Lu et al., 2025; Guo et al., 2024). This integration enables the dynamic dissection of yield formation mechanisms governed by “canopy physiological responses - multi-factor environmental feedback” interactions.

Crop yield formation is characterized as a complex nonlinear process influenced by multiple factors, including terrain, phenological resources, and soil nutrients. Machine learning methods are capable of fitting implicit relationships between multiple data resources and the combination of representative models with remotely sensed data, such as random forest (RF) and gradient boosted decision trees (GBDT), have demonstrated superiority in yield estimation (Paudel et al., 2023; Li et al., 2022; Lischeid et al., 2022). Features are extracted from input data by deep learning models through multi-layer implicit neural network structures; complex nonlinear relationships can thereby be fitted, and an advanced application of machine learning in the domain of feature extraction is thus demonstrated (Kamilaris and Prenafeta-Boldú (2018). Due to their capability in modeling intricate data relationships, deep learning techniques are widely acknowledged for their superior performance in natural language processing and crop classification, which has motivated researchers to focus extensively on the application of remote sensing data-driven deep learning models to crop yield estimation (Wang et al., 2022). Long Short-Term Memory (LSTM) structures were introduced by Lu et al (2024b) for maize yield estimation in northeastern China, and an effective method was provided to quantify the impacts of external factors on maize production (Lu et al., 2024b). To capture the spatio-spectral features and temporal dependencies in remote sensing images, Wang et al. (2023) proposed a multi-spatial image yield estimation method based on dimensionality reduction techniques and three-dimensional convolutional neural networks, which effectively improved the accuracy of crop yield estimation (Wang et al., 2023).Additionally, latent features in long-term sequential images are effectively captured by LSTM architectures, through which spectral, spatial, and temporal information across the entire crop growth cycle is extracted, thereby enhancing yield estimation accuracy. Temporal series data are accurately and interpretably modeled by Transformer architectures via their self-attention-based encoder-decoder framework without requiring recurrent units (Du et al., 2025). Although these two models are validated for their respective strengths in deep learning applications, the integration of Transformer and LSTM architectures with the mixture-of-experts (MoE) mechanism remains underexplored. The Mixture of Experts (MoE) is an ensemble learning framework that employs multiple specialized “expert” subnetworks, each trained to handle different parts of the input space or different subtasks. A gating network then adaptively weights the contributions of these experts for a given input, allowing the model to effectively tackle complex problems by dividing them into simpler, more manageable components (Li et al., 2025; Rajbhandari et al., 2022). Therefore, in the field of wheat yield estimation, the exploration of MoE models combining Transformer-LSTM architectures with spatiotemporal feature fusion is considered to hold substantial research value.

Hybrid models are increasingly applied across multiple domains (Sansana et al., 2021). A CNN-LSTM hybrid architecture was constructed by Oikonomidis et al (2022) for crop yield estimation, through which the combination of spatial feature extraction and temporal modeling was utilized, and estimation accuracy was enhanced (Oikonomidis et al, 2022). A Transformer-LSTM model was designed by Jiang et al. (2024) for spatiotemporal meteorological data prediction, and the superiority of hybrid architectures was validated (Jiang et al., 2024a). Through practical implementation, the integration of recurrent structures into Transformer frameworks is demonstrated to significantly enhance their capability in processing temporal series data.

Despite the excellent performance of deep learning models, their ‘black-box’ nature limits interpretability. *Post-hoc* interpretability methods aim to reveal model reasoning and enhance comprehensibility, though not primarily for performance improvement (Markus et al., 2021). As a prominent *post-hoc* explanation tool, Shapley Additive Explanations (SHAP) is employed in this study to quantitatively assess the contribution of individual variables to the yield estimation models.

Despite significant advancements in wheat yield estimation using multi-source remote sensing data, critical research gaps persist. Existing methods, including sophisticated deep learning techniques, often encounter difficulties in effectively integrating complex spatiotemporal data heterogeneity, dynamically adapting feature extraction and fusion strategies to diverse agricultural conditions (Zhu et al., 2018), and achieving a flexible learning balance between model complexity and estimation accuracy without resorting to overly rigid architectures (Hu et al., 2021). This highlights a pressing need for novel architectures capable of more adaptive information fusion and dynamic processing allocation to handle the multifaceted nature of yield formation. To address these specific limitations concerning spatiotemporal complexity, adaptive learning, and dynamic feature fusion, this study introduces a novel spatiotemporal fusion mixture-of-experts (STF-MoE) deep learning architecture. The proposed STF-MoE model is built upon a robust LSTM-Transformer hybrid framework and innovatively incorporates a heterogeneous mixture-of-experts mechanism. This mechanism is specifically designed to enhance spatiotemporal feature fusion capabilities by dynamically focusing on the most salient features and allocating specialized processing to different aspects of the input data, thereby tackling the multifaceted nature of yield formation. The model is applied to county-level wheat yield estimation, and the SHAP method is employed to interpret the estimation process and feature importance. The objectives are: (1) to develop the STF-MoE architecture and evaluate its performance against baseline models; (2) to validate the model’s robustness across different spatial scales and error magnitudes; and (3) to explore the interpretability of the STF-MoE model through analysis of selected test years.

## 2 Materials and methods

### 2.1 Materials

#### 2.1.1 Study area

**Figure 1:**
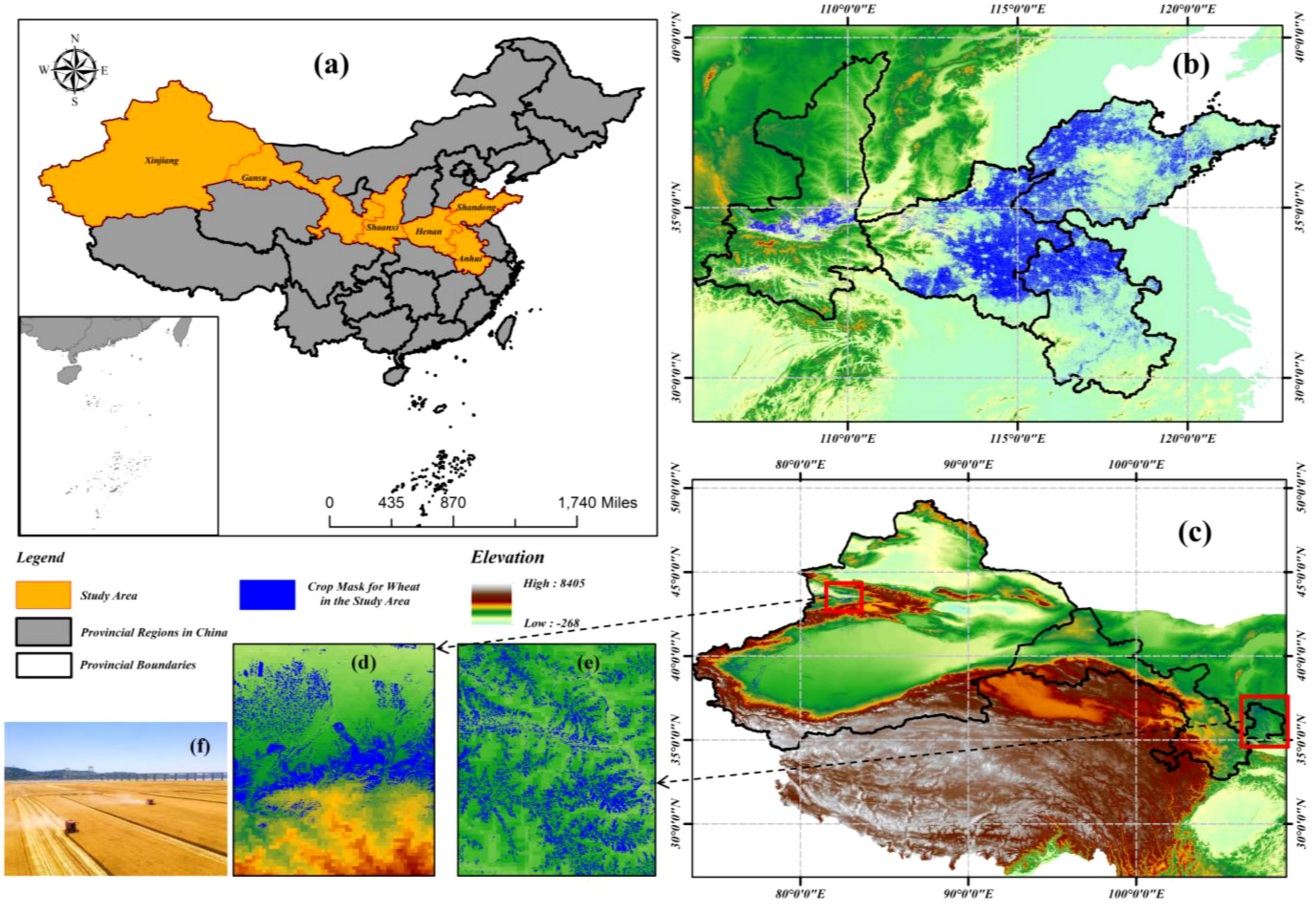
Hierarchical spatial distribution of Chinese wheat cultivation. **(a)** National–scale production zones. **(b)** Huang-Huai-hai Plain Wheat Region. **(c)** Wheat-growing areas in the arid and semi-arid regions of Northwest China. **(d)** High–density Xinjiang cultivation clusters. **(e)** Gansu Province Core Production Zones. **(f)** Field–scale characteristics in Huang-Huai-Hai Plain (Image credit: https://www.vcg.com/).

The study area is confined to major wheat-producing regions in mainland China, spanning a transitional ecological zone from subtropical monsoon to temperate continental arid climates. Six provincial-level administrative units are encompassed: Xinjiang Uygur Autonomous Region (southern Xinjiang oasis irrigation zone), Gansu Province (Hexi Corridor rainfed agriculture zone), Shaanxi Province (Guanzhong Plain winter wheat zone), Henan Province (core production area of Huang-Huai-Hai Plain), Anhui Province (Jiang-Huai watershed transition zone), and Shandong Province (high-yield intensive farming region of North China Plain), as illustrated in **Figure 1**. The Huang-Huai-Hai wheat belt and Northwest wheat belt, which are two of China’s three dominant wheat production zones, are covered by this region, accounting for 79.66% of the national wheat cultivation area. A representative ecological gradient is provided by the climatic zonation and topographic diversity across this region, serving as a scientific transect for investigating wheat ecophysiological adaptability (Zhao, 2010).

#### 2.1.2 Farmland data and wheat yield data

In this study, winter wheat yield statistics (unit: kg/ha) were obtained from county-/city-level statistical yearbooks published by provincial bureaus of China (2002–2021). Specifically, prefecture-level data were used for Shaanxi, Shandong, Anhui, and Henan provinces, while county-level data were applied to Gansu Province and Xinjiang Uygur Autonomous Region. After regional screening and integration, 25,200 records (covering 126 cities/counties) were included, ensuring data completeness across all years within the study area. Spatial distribution data of winter wheat were sourced from the “2001–2023 China Winter Wheat Planting Distribution Dataset at 30-m Resolution” (provided by the National Ecosystem Science Data Center, National Science & Technology Infrastructure of China: http://www.nesdc.org.cn), which was developed and continuously updated by Dong et al (2020); Dong et al, 2024), with related research contributions from Fu et al (2025b) (Dong et al., 2020, Dong et al, 2024; Fu et al., 2025b). The dataset was generated using a time-weighted dynamic time warping (DTW) method based on seasonal variation curve similarity, producing distribution maps that cover China’s major winter wheat planting areas (>99% of national coverage) with an overall accuracy of 91.6%. Data were formatted as GeoTIFF files under the Albers equal-area projection, represented as a binary classification (1 = winter wheat, 0 = non-winter wheat). This high-precision spatial data enabled precise delineation of winter wheat cultivation areas, effectively excluding interference from other crops and natural vegetation, thereby providing a reliable spatial foundation for subsequent analyses.

#### 2.1.3 Remote sensing data

##### 2.1.3.1 MODIS data and vegetation index

The MODIS Terra satellite MOD09A1 8-day composite surface reflectance product (500 m spatial resolution) was systematically analyzed to investigate spatiotemporal evolution patterns of surface reflectance characteristics across provinces, prefecture-level cities, and county-level units in China during 2001–2021. The observation period from September to June of the following year was selected to fully cover seasonal variation cycles, providing data support for understanding regional surface dynamics. Cloud-contaminated pixels were removed through StateQA band quality control to ensure data reliability. Four complementary vegetation indices were constructed based on red (620–670 nm, sur_refl_b01), near-infrared (841–876 nm, sur_refl_b02), and blue (459–479 nm, sur_refl_b03) band reflectance. The normalized difference vegetation index (NDVI) was used to characterize vegetation coverage and biomass (Bevz et al., 2024). The enhanced vegetation index (EVI) was designed with blue band and soil adjustment coefficients to reduce atmospheric and soil background interference (Woldemariam et al., 2024). The near-infrared reflectance of vegetation (NIRv) was generated by combining NDVI with near-infrared reflectance to optimize photosynthetic activity characterization (Lai et al., 2024).The green normalized difference vegetation index (GNDVI) was developed using green band reflectance to enhance chlorophyll sensitivity (Quille-Mamani et al., 2025).

The near-infrared reflectance of vegetation (NIRv) is defined as a recently developed vegetation index, through which interference from non-photosynthetic components on vegetation signals is structurally eliminated, with its calculation formula expressed as Equation 1:


(1) $$\begin{array}{c} \text{NIRv}={\text{ρ}}_{\text{NIR}}\times \text{NDVI}={\text{ρ}}_{\text{NIR}}\times \frac{{\text{ρ}}_{\text{NIR}}-{\text{ρ}}_{\text{Red}}}{{\text{ρ}}_{\text{NIR}}+{\text{ρ}}_{\text{Red}}} \end{array}$$

In this formulation, $\rho_{NIR}$ and $\rho_{Red}$ are defined as reflectance values of the near-infrared and red spectral bands, respectively. Background signal interference in vegetation photosynthetic activity estimation is structurally suppressed through this multiplicative formulation of NIRv, thereby enhancing the accuracy of vegetation photosynthetic capacity quantification. This approach is particularly applicable for monitoring vegetation seasonal dynamics and productivity variations.

Additionally, the MODIS Terra LAI/FPAR 8-day composite product (MOD15A2H, 500 m resolution) was integrated in this study, from which two key vegetation biophysical parameters, leaf area index (LAI) and fraction of absorbed photosynthetically active radiation (Fpar), were extracted. LAI is utilized to quantify vegetation canopy structural characteristics, while Fpar is employed to characterize vegetation light interception capacity within photosynthetically active radiation bands. Both parameters are retrieved through physical model inversion, establishing critical eco-physiological parameters for winter wheat growth assessment (Chen et al., 2017).

##### 2.1.3.2 Environmental variable data and related parameters

Meteorological variables were derived from ERA5-Land hourly reanalysis data, where monthly average relative humidity (RHum) and cumulative solar radiation (Rad) were computed. RHum was calculated from 2-meter air temperature and dewpoint temperature using the Magnus formula, while Rad was obtained by aggregating hourly data and converting it to megajoules per square meter (MJ/m²). Topographic data were integrated with the SRTM digital elevation model (DEM, 30-meter resolution), which was resampled to 500-meter resolution using bilinear interpolation, and slope (Slope) was calculated to quantify terrain effects. Soil organic carbon (SoC) content data were sourced from the OpenLandMap global open-access soil database (250-meter resolution), which was spatially resampled and aligned with MODIS data. All environmental variables were standardized to 500-meter spatial resolution and rigorously matched with vegetation index data across temporal (monthly scale) and spatial (county-level administrative boundaries) dimensions. Mask-based zonal statistical methods were applied to extract mean environmental parameters within masked cultivation areas, through which a multi-source dataset was constructed to support vegetation-climate-soil synergy mechanism analysis in wheat cropping systems.

### 2.2 Dataset preprocessing

**Figure 2:**
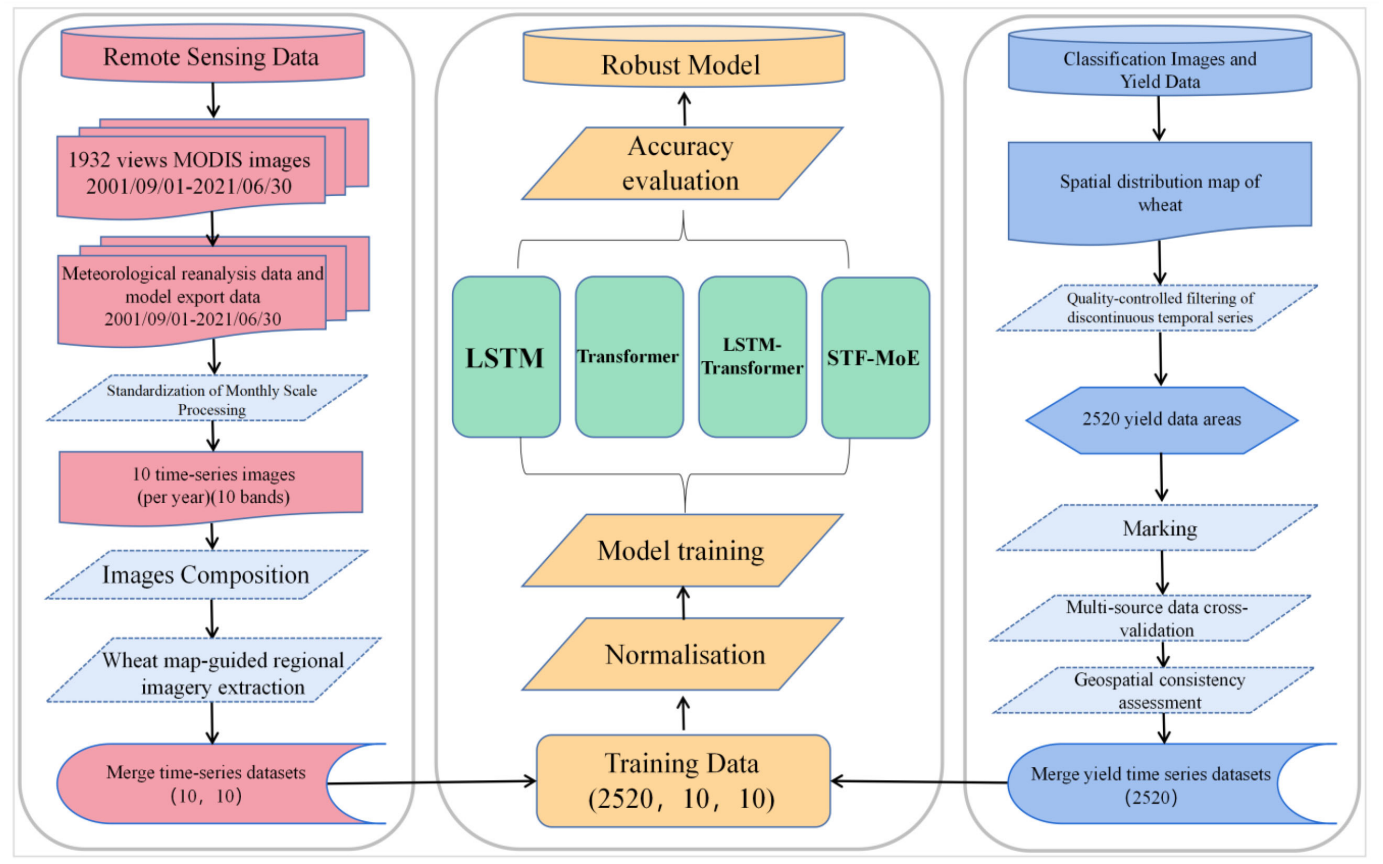
Flow chart of data processing and model training.

A county-level wheat yield estimation framework for China from 2002 to 2021 was constructed, with temporal coverage spanning the complete wheat growth cycle (September to June annually) to precisely capture nine key phenological stages. Dynamic impacts of climate change on provincial wheat cropping systems and phenological shifts were simultaneously considered. A multi-source data fusion strategy was adopted, under which MODIS remote sensing data, vegetation indices, and environmental parameters were systematically processed through spatial standardization (unified resampling to 500-meter resolution), temporal standardization (monthly scale), annualized masking based on wheat cultivation areas, and spatial aggregation to county-level administrative units. Rigorous data quality control methods were implemented to filter discontinuous or missing yield records, ensuring dataset completeness and reliability. Data processing workflows were executed on the Google Earth Engine cloud computing platform using pixel-level statistical methods and administrative boundary-based spatial aggregation, achieving efficient large-scale remote sensing data processing. Deep integration of remote sensing data with statistical records was realized through Python programming in the PyCharm integrated development environment with pandas library, laying a solid data foundation for high-precision wheat yield estimation models. Detailed specifications of the dataset are summarized in **Table 1**, and the integrated data processing and model training workflow is illustrated in **Figure 2**.

**Table 1:** Data sources.

Category	Variables	Temporal resolution	Spatial resolution	Time coverage	Data source
Wheat yield and planting area	Local and county–level production	Yearly	Local and county–level	2002–2021,From September to June of the following year	Statistical Yearbook of Each Province
	Planting area(The Cropland Data Layer )	Yearly	30m	2001–2021,From September to June of the following year	DOI:10.6084/m9.figshare.12003990.v2
MODIS data and vegetation index	Surface Reflectance (Red–Sur_Sefl–b01)	Daily	500m	2001–2021,From September to June of the following year	MOD09GA Version 6.1
	Vegetation index(NDVI,EVI, GNDVI)	16–day	500m	2001–2021,From September to June of the following year	MOD13A1 Version 6.1
	Vegetation index(NIRv)	Daily	500m	2001–2021,From September to June of the following year	Formula 1 can be calculated as
Environmental data	Photosynthesis–related Parameters(LAI,Fpar)	8–day	500 m	2001–2021,From September to June of the following year	MOD15A2H
	Meteorological Parameters(RHum, Rad)	1–hour	0.1°	2001–2021,From September to June of the following year	ECMWF ERA5–Land hourly data
	Topographic Parameters(DEM,Slope)	Yearly	30 m	2001–2021,From September to June of the following year	SRTMGL1 v3
	Soil Parameters(SoC)	Yearly	250 m	2001–2021,From September to June of the following year	SOL_ORGANIC–CARBON_USDA–6A1C_M/v02

### 2.3 Methods

#### 2.3.1 STF–MoE model

**Figure 3:**
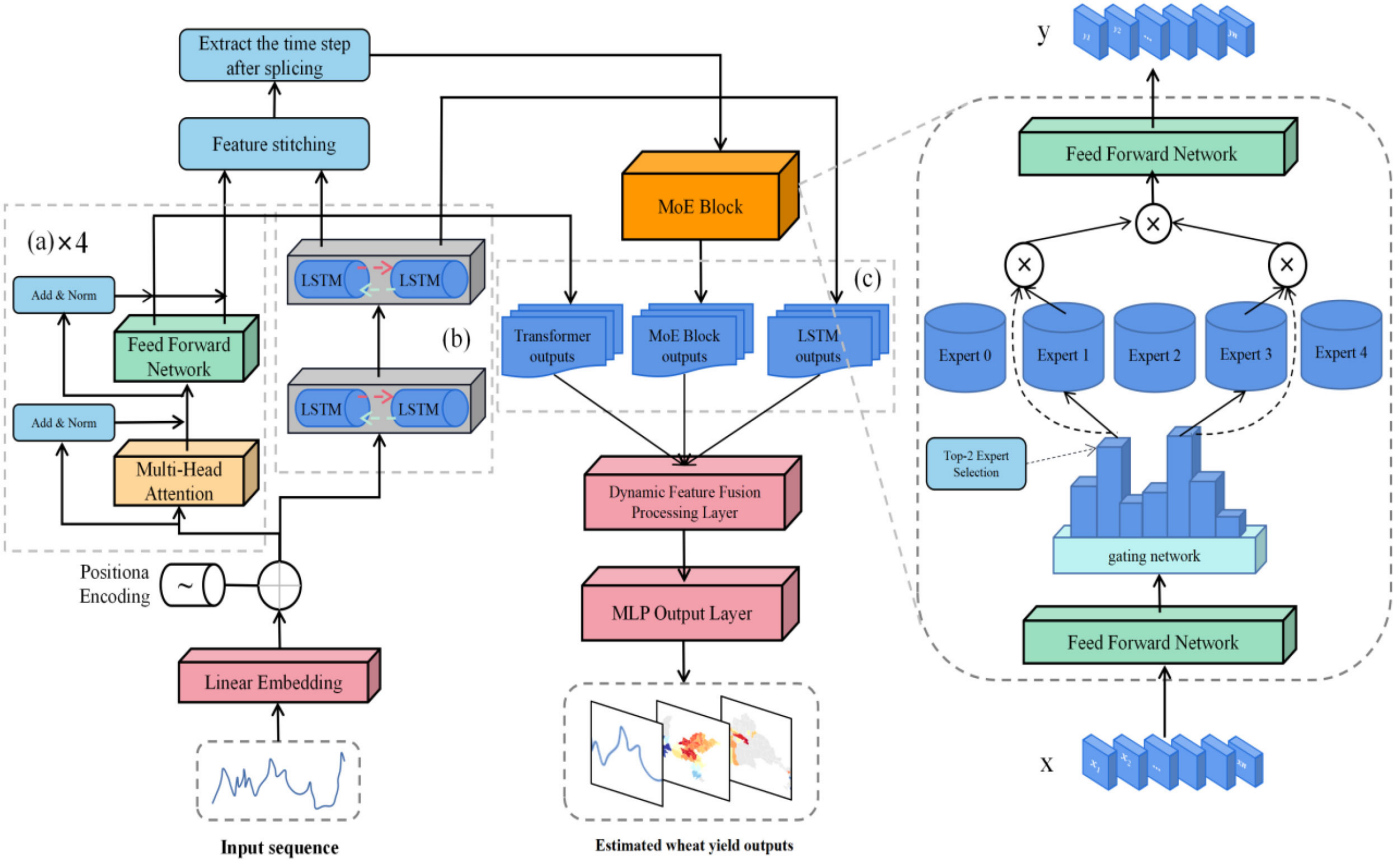
Architecture of the STF–MoE model: **(a)** Four–layer Transformer encoder; **(b)** Bidirec-tional LSTM module; **(c)** Feature fusion mechanism.

A deep learning model named Spatio–Temporal Fusion Mixture of Experts (STF-MoE)is proposed in this study, as illustrated in **Figure 3**. This architecture is improved from an LSTM–Transformer hybrid framework, where a heterogeneous mixture-of-experts mechanism is introduced to enhance spatiotemporal feature fusion capabilities. In the STF-MoE framework, remote sensing input data with an input feature dimension of 10 and a temporal step size of 10 are mapped to a high-dimensional representation space through a linear embedding layer, while temporal sequence information is preserved via sinusoidal positional encoding. Wherein a time step of 10 is employed, this represents feature inputs from 10 months which span the entire wheat growing sea-son; furthermore, an input feature dimension of 10 indicates that the model input comprises these 10 remote sensing feature variables. The model core is composed of two parallel branches: A Transformer branch with 4 encoder layers (8 attention heads per layer) is designed to efficiently capture long-range temporal dependencies. A bidirectional two-layer LSTM branch is constructed to precisely extract local contextual features and temporal dynamics. Outputs from both branches are concatenated and dynamically routed through an adaptive gating network to 5 structurally heterogeneous expert networks, each of which is equipped with distinct hierarchical architectures, activation functions, and regularization strategies to specialize in specific feature patterns. Expert weights are computed via a Softmax function in the gating mechanism, with the Top-2 experts being selected for feature processing to optimize computational resource allocation. Final yield estimation is generated through a three-stage feature fusion layer where MoE outputs, Transformer final states, and LSTM final states are integrated. A 512-dimensional fusion layer is followed by a 256-dimensional nonlinear mapping layer to produce high-precision wheat yield estimations. During the training phase, the Huber loss function is employed to balance outlier robustness and convergence speed. The Adam optimizer is configured with an initial learning rate of 0.0001, and a batch size of 32 is set to ensure GPU memory efficiency and gradient estimation stability over 600 training epochs. Gradient clipping is implemented with a threshold of 1.0 to prevent gradient explosion, while hierarchical Dropout (0.1–0.3) is applied to suppress overfitting. All feature standardization is rigorously performed using MinMaxScaler parameters fitted from the training set, ensuring model generalizability.

#### 2.3.2 Mixture of experts architecture

In the STF-MoE model architecture, a mixture-of-experts (MoE) framework is employed with an efficient conditional computation paradigm, where adaptive feature processing of inputs is achieved through a dynamic routing mechanism. The MoE module receives the spatiotemporal feature representations extracted by the Transformer and bidirectional LSTM branches, and a comprehensive input is formed through the concatenation of these representations. Specifically, global dependencies are captured by the Transformer branch via multi-head self-attention mechanisms, while temporal-local features are extracted by the bidirectional LSTM branch through recurrent architectures. Output features from these two components are concatenated along the hidden dimension, generating the input feature representation $h\in R^{b\times t\times 2d}$ , where b denotes batch size, t represents sequence length, and d corresponds to the model’s hidden dimension.

##### 2.3.2.1 Gating network design

The core of the MoE architecture is implemented as a gating network, through which expert weights are dynamically allocated. The gating network is composed of two fully connected layers, between which the GELU activation function is employed to enhance nonlinear representation capabilities. A probability distribution of experts is generated via the Softmax function. Formally, the computational process of the gating network is defined by Equation 2:


(2) $$\begin{array}{c} \text{g}=\text{Softmax}\left({\text{W}}_{2}\cdot \text{GELU}\left({\text{W}}_{1}\cdot {\text{h}}_{\text{avg}}+{\text{b}}_{1}\right)+{\text{b}}_{2}\right) \end{array}$$

Where $h_{avg}\in R^{b\times 2d}$ is obtained by averaging the input features h along the temporal dimension. The weight matrices of the fully connected layers are represented as $W_{1}\in R^{2d\times 256}$ and $W_{2}\in R^{256\times 5}$ , while the bias vectors are defined as $b_{1}\in R^{256}$ and $b_{2}\in R^{5}$ . The gating network output $g\in R^{b\times 5}\;$ is interpreted as assignment probabilities of each sample to the five experts.

##### 2.3.2.2 Sparse activation mechanism

To enhance computational efficiency and mitigate overfitting risks, a sparse activation strategy is employed in the model, where only the Top-2 experts with the highest probabilities in the gating network output are selected for computation. For each sample i, the gating output $g_{i}\in R^{5}$ is sorted to obtain the top k=2 expert indices $\epsilon_{i}=\left\{e_{i_{1}},e_{i_{2}}\right\}\;$ and corresponding weights $w_{i}=\left\{w_{i_{1}},w_{i_{2}}\right\}$ , where $w_{i_{j}}=g_{i}\left[e_{i_{j}}\right]$ .

##### 2.3.2.3 Expert processing and weighted fusion

The implementation of the heterogeneous expert pool, which comprises multiple expert subnetworks, each with distinct architectural designs to foster specialization on varied data patterns, is governed by two critical phases: output standardization and dynamic fusion. To ensure compatibility among ex-pert network outputs, an Expert Adapter layer is introduced, through which output dimensions are standardized to d. The transformation Equation 3 is defined as:


(3) $$\begin{array}{c} {\text{o}}_{{\text{i}}_{\text{j}}}={\text{W}}_{\text{adapt}}\cdot \text{Exper}{\text{t}}_{{\text{e}}_{{\text{i}}_{\text{j}}}}\left({\text{h}}_{\text{i}}\right)+{\text{b}}_{\text{adapt}} \end{array}$$

In this formulation, $o_{i_{j}}\in R^{d}$ is defined as the output vector of the i-th input sample processed and adapted by the j-th selected expert, with dimensionality d. This vector is represented as a standardized feature representation for subsequent fusion. $W_{adapt}\in R^{d_{expert}\times d}$ and $h_{i}\in R^{t\times 2d}$ are defined, where $h_{i}\in R^{t\times 2d}$ is interpreted as the spatiotemporal feature representation of the i-th input sample, with dimensions corresponding to sequence length t and feature dimension 2d. It is derived from the concatenation of features extracted by the Transformer and bidirectional LSTM branches. The adapter layer is designed to ensure dimensional consistency among heterogeneous expert outputs, establishing the foundation for dynamic fusion. Through its linear transformation properties, feature information extracted by expert networks is preserved, while adaptive adjustments to diverse expert outputs are enabled via learnable parameters $W_{adapt},b_{adapt}$ .

After output standardization, expert weights are calculated by the gating network based on input features, and outputs from selected experts are dynamically fused through the sparse activation strategy (Top-2 selection) to generate the final integrated feature representation. The mathematical expression is given by Equation 4:


(4) $$\begin{array}{c} {\text{o}}_{\text{i}}=\sum_{\text{j}=1}^{2}{\text{w}}_{{\text{i}}_{\text{j}}}\cdot {\text{o}}_{{\text{i}}_{\text{j}}} \end{array}$$

Where $w_{i_{j}}\in \left[0,1\right]$ is assigned by the gating network as the weight of the j-th selected expert for the i-th input sample, indicating the contribution level of this expert to the final output. Expert weights are calculated from the Softmax output of the gating network and are filtered through the Top-2 strategy to ensure j=1,2. The weight calculation of the gating network is dependent on the global representation of input features h_i_ (typically obtained through temporal dimension averaging). Expert assignment probabilities are generated through a two-layer fully connected network and the Softmax function, after which the Top-2 experts and their corresponding weights are selected.

#### 2.3.3 Heterogeneous expert pool design

In the STF-MoE model, a Heterogeneous Expert Pool is implemented, composed of five architecturally distinct neural network experts. Each expert is optimized for specific temporal feature patterns through specialized structural design. This architectural heterogeneity is demonstrated to significantly enhance the model’s representational capacity and generalization performance, enabling it to effectively process diverse features in complex temporal data.

Five expert networks within the Heterogeneous Expert Pool are differentiated in terms of network depth, hidden layer dimensionality, activation function selection, and regularization strategies. Such architectural diversity is designed to enable the model to adapt to intrinsic complexity and variability in temporal data, while being governed by the gating network mechanism to allocate the most suitable expert combinations for distinct input features. The heterogeneous expert design is engineered to enhance the model’s capability to capture both long- and short-term dependencies as well as nonlinear patterns in temporal data, thereby strengthening its adaptability when processing non-stationary temporal data. The Heterogeneous Expert Pool is composed of the following five expert net-works:

##### 2.3.3.1 A two-layer GELU-activated expert is constructed

This expert is composed of two hidden layers, each of which is followed by GELU activation functions and Dropout layers. Its computational process is formulated as Equation 5:


(5) $$\begin{array}{c} \text{Exper}{\text{t}}_{0}\left(\text{x}\right)=\text{Dropout}\left(\text{GeLU}\left({\text{W}}_{2}\cdot \text{Dropout}\left(\text{GeLU}\left({\text{W}}_{1}\cdot \text{x}+{\text{b}}_{1}\right)\right)+{\text{b}}_{2}\right)\right) \end{array}$$

Where $W_{1},W_{2}\in R^{2d\times d}$ are defined as weight matrices, b_1_ is specified as the first-layer bias vector with dimensionality matching the output layer, where a translational shift is introduced to enhance linear transformation flexibility. b_2_ is defined as the second-layer bias vector, dimensionally aligned with the output of W_2_. Dropout probabilities are set to 0.2 and 0.1, respectively. This expert is designed to effectively extract medium complexity temporal features. The choice of a two-layer architecture provides a balance between representational power and computational cost, making it suitable for capturing moderately complex temporal patterns without excessive parameters. The GELU activation function is employed for its smooth, nonlinear characteristics, which, unlike ReLU, allows for negative values and provides a probabilistic interpretation, potentially leading to better performance in modeling continuously varying patterns and more complex functions. Its nonlinearities help in learning intricate data relationships. Furthermore, the inclusion of Dropout with probabilities of 0.2 and 0.1 in respective layers serves as a crucial regularization technique, preventing overfitting by randomly deactivating neurons during training, thereby promoting the learning of more robust and independent features and enhancing the model’s generalizability across diverse wheat yield temporal datasets. This expert system is typically applicable to the majority of general feature extraction tasks for wheat yield.

##### 2.3.3.2 A wide-layer ReLU-activated expert is constructed

This expert is composed of a wide hidden layer of dimensionality 2d and is activated by the ReLU function, with its computational process defined in Equation 6:


(6) $$\begin{array}{c} \text{Exper}{\text{t}}_{1}\left(\text{x}\right)={\text{W}}_{2}\cdot \text{Dropout}\left(\text{ReLU}\left({\text{W}}_{1}\cdot \text{x}+{\text{b}}_{1}\right)\right)+{\text{b}}_{2} \end{array}$$

Where $W_{1}\in R^{2d\times 2d}\;$ and $W_{2}\in R^{2d\times d}$ are defined as weight matrices, and the Dropout probability is set to 0.3. This expert is specifically designed to capture complex nonlinear patterns in high-dimensional temporal data. The wide-layer architecture, with a hidden layer dimensionality of 2d, significantly enhances the network’s capacity to learn a richer set of features and their interactions simultaneously from high-dimensional inputs. This breadth allows for more diverse feature combinations to be explored in a single layer, which is particularly beneficial for capturing complex, nonlinear relationships without resorting to excessive depth that could increase training difficulty. The Rectified Linear Unit (ReLU) activation function is chosen for several key advantages in this context: its sparsity (outputting zero for negative inputs) helps to reduce computational load and can lead to more disentangled representations; its nonsaturating nature in the positive domain alleviates the vanishing gradient problem, facilitating faster and more effective training, especially when combined with the wide architecture. The inherent computational efficiency of ReLU, coupled with the enhanced feature representation capabilities of the wide design, makes this expert adept at modeling intricate temporal dynamics. A Dropout probability of 0.3 is applied to mitigate the risk of overfitting, which can be more pronounced in wider networks due to the increased number of parameters.

##### 2.3.3.3 A narrow-layer SiLU-activated expert is constructed

This expert is composed of a narrow hidden layer with dimensionality d/2 and is activated by the SiLU function, as defined in Equation 7:


(7) $$\begin{array}{c} \text{Exper}{\text{t}}_{2}\left(\text{x}\right)=\text{SiLU}\left({\text{W}}_{2}\cdot \text{Dropout}\left(\text{SiLU}\left({\text{W}}_{1}\cdot \text{x}+{\text{b}}_{1}\right)\right)+{\text{b}}_{2}\right) \end{array}$$

Where $W_{1}\in R^{2d\times 2d},W_{2}\in R^{2d\times d}$ are defined as weight matrices, and the Dropout probability is set to 0.1. This expert is specialized in extracting local details and subtle fluctuations in temporal data. The narrow-layer design, with a hidden layer dimensionality of d/2, deliberately constrains the model’s capacity, forcing it to focus on more fine-grained, localized patterns and subtle variations within the temporal data rather than global, complex features. This reduction in dimensionality also contributes to lower computational complexity and a reduced risk of learning spurious correlations from noise, making it efficient for dissecting intricate local dynamics. The Sigmoid Linear Unit (SiLU, also known as Swish) activation function is selected for its unique properties: it is a smooth, nonmonotonic function that often outperforms ReLU, especially in deeper networks, by combining the benefits of linearity for large positive inputs (avoiding saturation) with a selfgating mechanism that allows for better gradient flow and expressive power. This enables the network to capture more nuanced feature variations and subtle temporal fluctuations effectively. The low Dropout probability of 0.1 is appropriate for this narrower architecture, providing regularization without overly restricting its learning capacity for these detailed features.

##### 2.3.3.4 A single-layer GELU-activated expert is constructed

The single-layer GELU-activated expert is composed of a single fully connected layer followed by the GELU activation function. Its mathematical representation is formulated as Equation 8:


(8) $$\begin{array}{c} \text{Exper}{\text{t}}_{3}\left(\text{x}\right)=\text{GeLU}\left({\text{W}}_{1}\cdot \text{x}+{\text{b}}_{1}\right) \end{array}$$

Where $W_{1}\in R^{2d\times d}$ is defined as the weight matrix. This streamlined architecture is designed to prioritize computational efficiency and is recommended for rapid processing of linear or weakly nonlinear features, such as trend extraction tasks in stationary time series. The fundamental advantage of this expert lies in its extreme simplicity and computational efficiency, stemming from its single fully connected layer. This minimal architecture makes it exceptionally fast for processing large volumes of data where only linear or very simple nonlinear transformations are required. The GELU activation function is again employed for its smooth nonlinearity, offering a slight expressive advantage over a purely linear model while retaining high computational speed. This design is particularly well-suited for tasks like extracting dominant trends or baseline signals from stationary or near stationary time series, where complex feature interactions are not the primary concern, and quick, efficient processing is paramount.

##### 2.3.3.5 A three-layer ReLU-activated expert is constructed

The three-layer ReLU-activated expert is structured as a deep network architecture specifically designed to capture highly complex temporal features. Each layer is followed by a ReLU activation function and Dropout regularization. Its mathematical representation is expressed in Equation 9:


(9) $$\begin{array}{c} {\text{Expert}}_{4}\left(\text{x}\right)={\text{W}}_{3}\cdot \text{Dropout}\left(\text{ReLU}\left({\text{W}}_{2}\cdot \text{Dropout}\left(\text{ReLU}\left({\text{W}}_{1}\cdot \text{x}+{\text{b}}_{1}\right)\right)+{\text{b}}_{2}\right)\right)+{\text{b}}_{3} \end{array}$$

Where $\;W_{1},W_{2}\in R^{2d\times \left(\frac{d}{2}\right)}\;$ (assuming W_3_ maps from d/2 to d) are specified as weight matrices with hidden layers of dimensionality d/2, and the Dropout probability is configured at 0.2 for each Dropout layer. The primary strength of this expert lies in its three-layer deep architecture. This increased depth allows the network to learn a hierarchical representation of features, where each successive layer builds more abstract and complex concepts from the outputs of the previous layer. This hierarchical processing is crucial for capturing highly intricate temporal patterns and long-term dependency relationships, such as those arising from cumulative climate effects or complex multivariable interactions influencing wheat growth, which shallower networks might fail to model adequately. The use of ReLU activation in each layer helps to mitigate the vanishing gradient problem often encountered in deeper networks, promoting stable and efficient training. The consistent application of Dropout with a probability of 0.2 after each ReLU layer provides robust regularization, which is particularly important in deeper architectures to prevent overfitting by discouraging complex co-adaptations of neurons and ensuring that the learned hierarchical features are generalizable.

#### 2.3.4 Comparative theoretical analysis

The estimation of wheat yield is challenged by complex temporal dependencies in time series, diverse characteristics of multi-source data, and significant variations across growth stages. Traditional deep learning models are observed to exhibit certain limitations (Ishaq et al., 2025). While Long Short-Term Memory (LSTM) networks are capable of capturing temporal dependencies, their recursive computation demonstrates low efficiency when processing long-sequence data and is prone to gradient vanishing issues (Safwan Mahmood et al., 2023). This architecture is found to be insufficient for simultaneously accommodating distinct features such as sudden precipitation events and periodic temperature patterns, nor can it perform parameterized adjustments according to different phenological phases. Transformer models, though demonstrating superior performance in global dependency modeling, are limited in their ability to characterize gradual developmental changes and short-term meteorological events through their self-attention mechanisms (Li and Lu (2023). Furthermore, their uniform feed-forward network structure is proven inadequate for effectively processing heterogeneous data sources including vegetation indices, meteorological, and soil data. Existing LSTM-Transformer hybrid models are constrained by inflexible fusion mechanisms, with particular deficiencies identified in their capacity to dynamically adjust feature weights according to data heterogeneity (Li et al., 2024).

This study proposes an improved model named STF–MoE, which addresses the aforementioned challenges through innovative architectural design. The model is constructed with a Transformer branch that enables parallel processing of entire growing season data to capture long-range temporal dependencies, while a bidirectional LSTM branch is incorporated to enhance local growth dynamics modeling and precisely describe continuous physiological change processes. A heterogeneous expert system is implemented through structurally differentiated neural networks–including deep-narrow networks with GELU activation and wide-shallow networks based on ReLU–to specifically handle multi-source agricultural features, achieving adaptive modeling for critical growth stages. The core innovation lies in the introduced MoE gating mechanism, which dynamically activates the most suitable combination of experts according to spatiotemporal patterns of input features. Through an adaptive weight matrix that integrates multi-source features, the model optimizes feature processing strategies for diverse ecological environments and climatic conditions.

The theoretical advantages of STF–MoE are primarily demonstrated in the following aspects: First, the TopK sparse gating mechanism enables dynamic allocation of computational resources, where computational complexity is adjusted according to the significance of different phenological stages. Second, the heterogeneous expert design eliminates structural homogeneity constraints inherent in conventional models, allowing adaptation to distinct feature distributions across meteorological data, soil parameters, and vegetation indices. Finally, the multi-level spatiotemporal feature fusion strategy enhances the model’s representational capacity for complex spatiotemporal interaction pat-terns, facilitating the interpretation of nonlinear relationships among climate variations, soil conditions, and vegetation indices. By integrating the global modeling capability of Transformers, sequential memory characteristics of LSTM, and adaptive feature extraction mechanisms of heterogeneous expert systems, STF–MoE provides a novel solution for wheat yield estimation. However, given the model’s architectural complexity, critical aspects such as estimation accuracy still require validation through further comparative experiments to ensure applicability within specific agricultural ecosystems and multi-source datasets, as well as to verify model interpretability.

#### 2.3.5 Comparative experiments

Building upon the theoretical analysis of the STF–MoE model presented in Section 2.3.4,this section is designed to systematically evaluate its performance in wheat yield estimation tasks. To comprehensively validate the effectiveness of the STF–MoE model, comparative experiments were designed by selecting representative time series processing models including LSTM, Transformer and LSTM–Transformer as benchmarks. These models collectively embody different technical approaches for temporal data processing, establishing a multidimensional reference framework for performance evaluation of the STF–MoE model.

The experiment employed a unified dataset for model training and performance evaluation to ensure fairness and reliability in comparative analysis. During the data preprocessing phase, standardized processing procedures including missing value handling, feature normalization, and temporal feature extraction were implemented by this study. Specifically, the application of MinMaxScaler normalization to features combined with sliding window techniques for time-series feature extraction not only standardized input formats across all models but also significantly enhanced estimation accuracy and stability in wheat yield estimation. These preprocessing measures were particularly designed to address inherent characteristics of agricultural data such as seasonal fluctuations and uncertainty characteristics.

All deep learning models were configured with identical hyperparameter settings to maintain experimental consistency: a learning rate of 0.0001, Huber loss function, and Adam optimization algorithm. This configuration ensured uniformity in experimental conditions while effectively addressing potential outlier issues in agricultural datasets. The selection of the Huber loss function was determined through comprehensive consideration of both mean squared error (MSE) and mean absolute error (MAE) advantages, achieving optimal balance between sensitivity to normal data distributions and robustness against outliers. The adaptive learning rate properties inherent in the Adam optimizer were specifically employed to handle sparse gradients and nonstationary characteristics commonly observed in agricultural time-series data, thereby guaranteeing stable estimation performance under complex and variable agronomic conditions.

#### 2.3.6 Performance evaluation

To systematically validate the STF-MoE model, a dual evaluation strategy was adopted in this study. Firstly, three years—2002 (representing the early dataset phase), 2013 (an extreme weather-impacted year), and 2021 (the most recent year)—were selected as independent test sets, while data from the remaining years were partitioned into training and validation sets at an 8:2 ratio for comprehensive evaluation. Secondly, a year-by-year rolling test protocol was designed to rigorously assess the model’s temporal generalizability: each year from 2002 to 2021 was individually designated as a test set, and training/validation sets were constructed following a temporally sensitive principle (e.g., data from 2002–2015 and 2017–2021 were used to predict 2016; data from 2003–2021 were used to predict 2002, etc.), maintaining an 8:2 ratio to evaluate performance across varying temporal spans (Johnson, 2014). To holistically evaluate the model’s yield estimation capability, three key metrics were selected: the coefficient of determination (R²), root mean square error (RMSE), and mean absolute error (MAE). Optimal estimation performance is indicated when R² approaches 1, while RMSE and MAE approach 0. Computational formulas for these metrics are detailed in Equations 10–12.


(10) $$\begin{array}{c} {\text{R}}^{2}=1-\sum_{\text{i}=1}^{\text{n}}{\left({\text{y}}_{\text{i}}-\hat{{\text{y}}_{\text{i}}}\right)}^{2}/\sum_{\text{i}=1}^{\text{n}}{\left({\text{y}}_{\text{i}}-\bar{\text{y}}\right)}^{2} \end{array}$$

(11) $$\begin{array}{c} \text{RMSE}=\sqrt{\left(\sum_{\text{i}=1}^{\text{n}}{\left({\text{y}}_{\text{i}}-\hat{{\text{y}}_{\text{i}}}\right)}^{2}\right)/\text{n}} \end{array}$$

(12) $$\begin{array}{c} \text{MAE}=\frac{1}{\text{n}}\sum_{\text{i}=1}^{\text{n}}\left|{\text{y}}_{\text{i}}-\hat{{\text{y}}_{\text{i}}}\right| \end{array}$$

where n is defined as the total sample size; yi and ybreak poi denote the actual and estimated values respectively; represents the mean value of the actual observations.

## 3 Results

### 3.1 Exploratory feature data analysis

**Figure 4:**
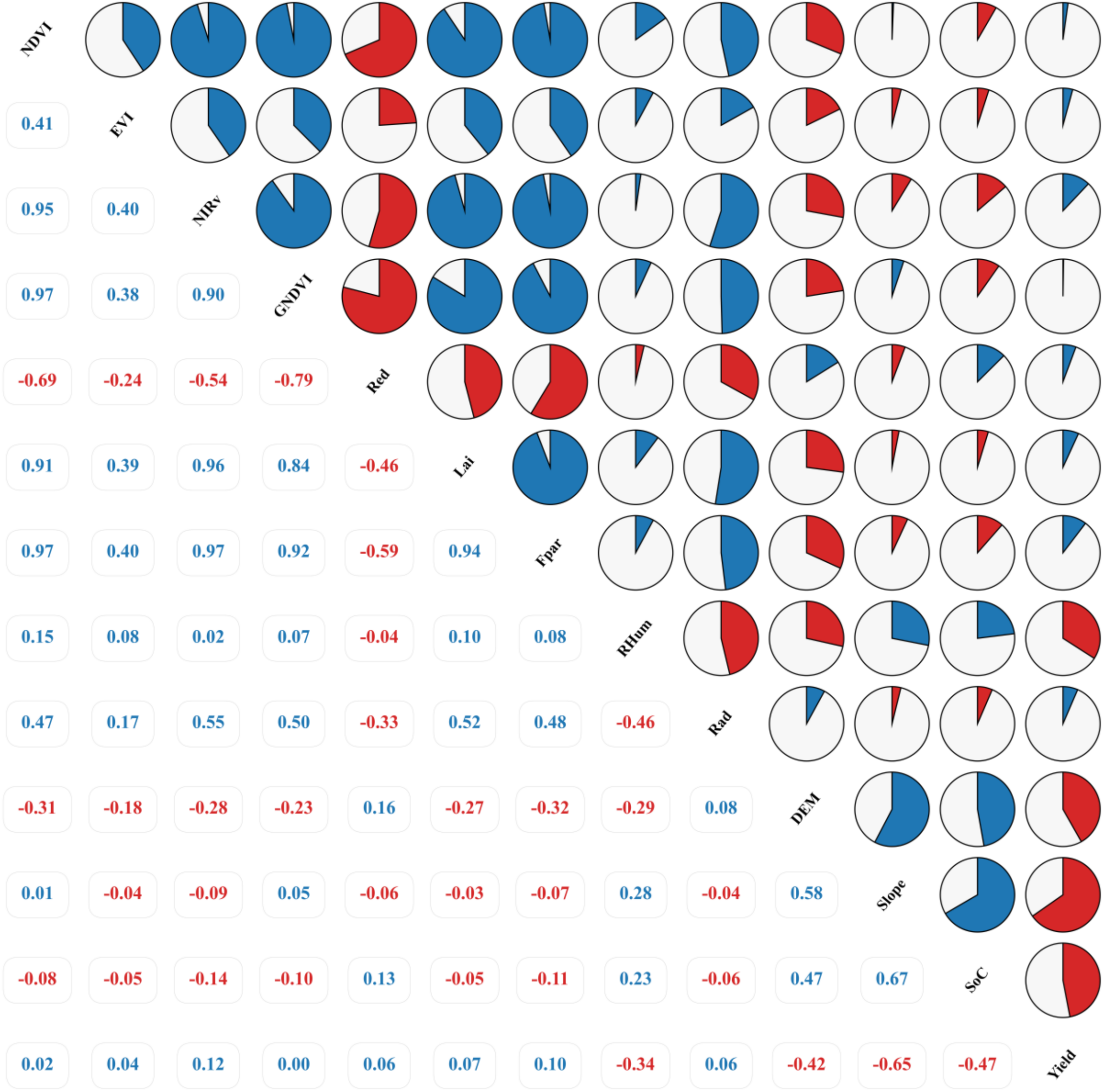
illustrates the correlation heatmap between significant variables and crop yield. Blue and red colors denote positive and negative correlations, respectively, with the arc segment size quantitatively reflecting the magnitude of correlation strength.

To guide variable selection for robust yield modeling, correlations between wheat yield and a series of predictors (remote sensing data, biophysical parameters, and environmental topography) in the study area during 2002–2021 were analyzed, as presented in **Figure 4**, with the dual objectives of quantifying linear associations and diagnosing collinearity issues. A novel visualization methodology was subsequently implemented to enhance the interpretative representation of these analytical results (Jiang et al., 2024b). Based on this analysis and collinearity assessment, 10 predictors were selected for subsequent modeling: NDVI, EVI, NIRv, Red, LAI, Fpar, RHum, Rad, DEM, and SoC, while GNDVI and Slope were excluded due to collinearity risks and model complexity concerns.

The correlation analysis (**Figure 4**) revealed that common vegetation indices (NDVI, EVI, NIRv) and biophysical parameters (LAI, Fpar) exhibited generally weak linear correlations with wheat yield (r-values ranging from 0.02 to 0.12). This suggests that single-temporal vegetation growth metrics may inadequately capture key yield-determining variations, or the relationships between these metrics and yield are nonlinear, potentially influenced by phenological stages, saturation effects, or other factors. This observation of weak linear correlations is not uncommon in complex agroecosystems, as reported in other studies (Lu et al., 2024c; Cao et al., 2021). Such findings often highlight that while direct linear relationships may be limited, these remote sensing variables still encapsulate crucial information about crop status that can be effectively harnessed by nonlinear models capable of discerning more intricate patterns and cumulative effects throughout the growing season. Notably, GNDVI showed near-zero linear correlation with yield (r≈0.00) and was highly collinear with other vegetation indices (e.g., NDVI, r=0.97) and biophysical parameters (e.g., Fpar, r=0.92; LAI, r=0.84). Therefore, to avoid multicollinearity and enhance model parsimony and robustness, GNDVI was excluded from subsequent modeling. In contrast, certain environmental and topographic factors demonstrated stronger associations, predominantly negative. Elevation (DEM, r=–0.42) and soil organic carbon (SoC, r=–0.47) showed moderate negative correlations with yield, likely associated with insufficient accumulated temperature in high-altitude regions/shortened growing seasons and specific soil/environmental constraints, respectively. The negative correlation of relative humidity (RHum, r=–0.34) may reflect disease risks induced by high moisture. Slope exhibited the strongest negative correlation (r=–0.65), indicating significantly lower yields in steep areas, potentially due to soil erosion, thinner soil layers, or cultivation management challenges. However, Slope also displayed moderate positive correlations with DEM (r=0.58) and SoC (r=0.67). Given that Slope’s effects may be partially mediated by DEM and SoC, and its strong correlation could introduce model complexity and overfitting risks, Slope was excluded from subsequent training to prioritize a generalized model focusing on vegetation physiology, soil properties, and macro-topographic influences. Red reflectance (Red, r=0.06) and radiation (Rad, r=0.06) showed negligible linear relationships with yield.

By comprehensively evaluating the correlation strength and collinearity of variables—particularly identifying the potential risks posed by Slope and GNDVI—subsequent modeling prioritized DEM, SoC, and RHum, which demonstrated moderate associations and relative independence, to construct a model emphasizing vegetation physiology, soil attributes, and macro-topographic impacts.

### 3.2 Accuracy comparison results: STF–MoE vs. baseline models

In the wheat yield estimation task, the STF-MoE model demonstrated its performance through a dual evaluation strategy. Core data revealed that during the year-by-year rolling tests from 2002 to 2021, the STF-MoE achieved a mean R² of 0.8712 and a mean RMSE of 529.0713 kg/ha (**Supplementary Tables S1**, **S2**). In comparison, the Transformer model yielded a mean R² of 0.8544 and a mean RMSE of 537.5832 kg/ha, while the LSTM model produced a mean R² of 0.8448 and a mean RMSE of 586.1364 kg/ha. This indicates that the STF-MoE exhibited an overall advantage, reducing the mean RMSE by 57 kg/ha (9.7%) relative to the LSTM.

**Figure 5:**
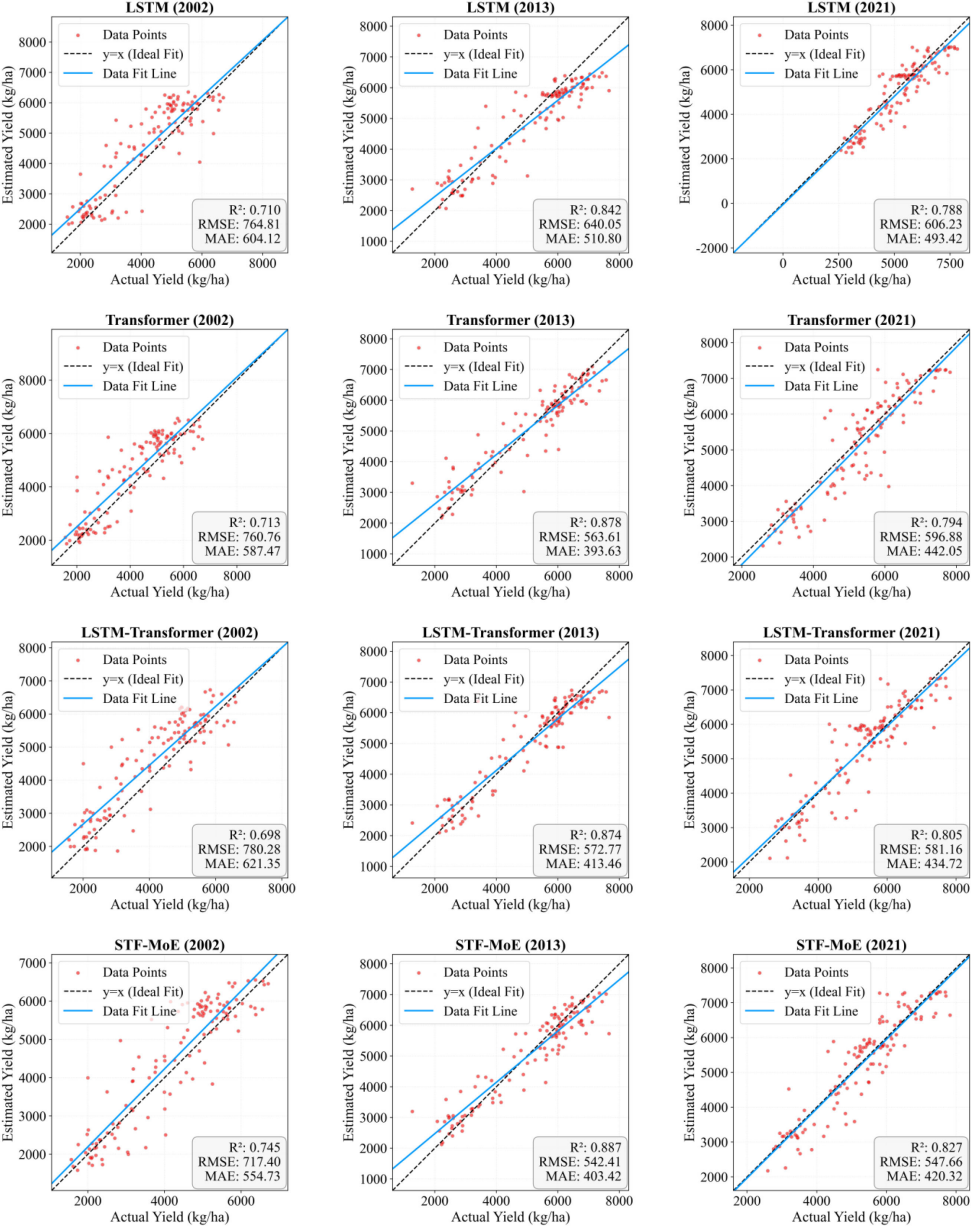
Scatter plots of estimated versus actual yields. Dashed lines represent regression lines (best-fit), and solid lines denote trend lines. The distribution of estimation scatters for four models across three independent test years is shown.

In evaluations across three independent test years (2002, 2013, 2021) (**Table 2**), the STF-MoE demonstrated relative superiority or competitive performance in R² and RMSE metrics: 2002 (R²: 0.745, RMSE: 717.4 kg/ha), 2013 (R²: 0.887, RMSE: 542.4 kg/ha), and 2021 (R²: 0.827, RMSE: 547.7 kg/ha). For instance, in 2013, its R² exceeded that of the Transformer (0.878), and its RMSE was lower than the Transformer’s (563.6 kg/ha). Scatter plots in **Figure 5** showed that predicted values from all models were generally distributed along the ideal fit line with actual values, though varying degrees of dispersion were observed. Notably, the STF-MoE also exhibited deviations from the ideal line, particularly in high-yield and low-yield regions. Year-by-year rolling test results (**Supplementary Tables S1**, **S2**) further revealed that the R² of STF-MoE fluctuated from a minimum of 0.7449 (2002) to a maximum of 0.9230 (2009), while RMSE varied from ~439.1 kg/ha (2009) to ~717.4 kg/ha (2002).

**Table 2:** Performance comparison of STF-MoE and baseline models on the 2002, 2013, and 2021 test sets.

Model	2002			2013			2021		
	RMSE(kg ha^–1^)	R^2^	MAE(kg ha^–1^)	RMSE(kg ha^–1^)	R^2^	MAE(kg ha^–1^)	RMSE(kg ha^–1^)	R^2^	MAE(kg ha^–1^)
Transformer	760.8	0.713	587.5	563.6	0.878	393.6	596.9	0.794	442.1
LSTM	764.8	0.710	604.1	640.1	0.842	510.8	606.2	0.788	493.4
LSTM–Transformer	780.3	0.698	621.4	572.8	0.874	413.5	581.2	0.805	434.7
STF–MoE	717.4	0.745	554.7	542.4	0.887	403.4	547.7	0.827	420.3

Collectively, the STF-MoE model demonstrated its capability in wheat yield estimation through superior mean performance metrics (mean R²: 0.8712; mean RMSE: 529.0713 kg/ha) and robust performance in specific independent years (e.g., R² = 0.887 in 2013). However, interannual performance variability was observed, with the R² varying by ~0.18 and RMSE fluctuating by nearly 280 kg/ha across years. Concurrently, scatter plots visually confirmed that predicted values exhibited notable deviations from actual values at yield extremes.

### 3.3 Yield estimation map of the STF-MoE model

**Figure 6:**
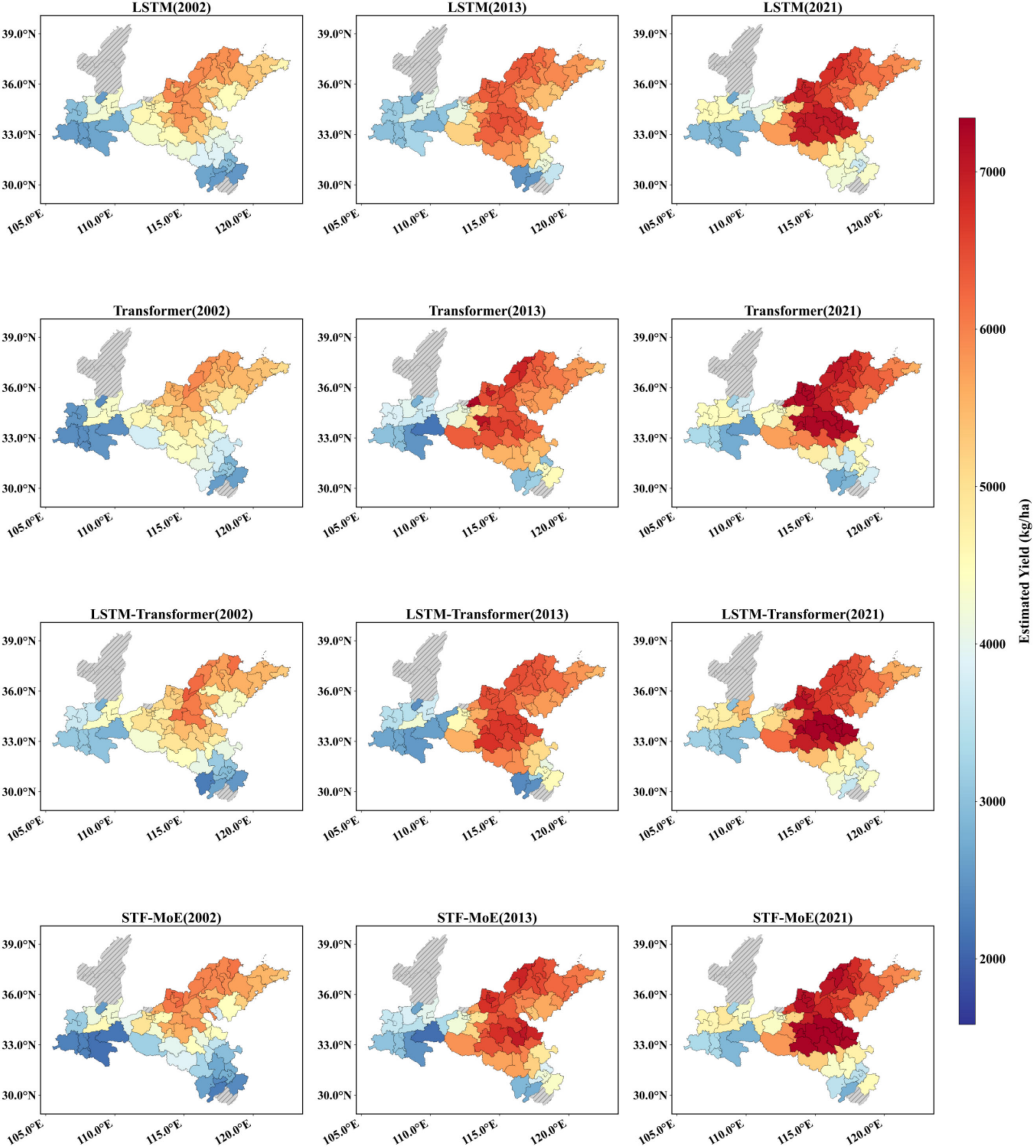
Prefecture-level yield estimation map. Red indicates high yields, and blue indicates low yields. The spatial distribution of estimations from four models across three independent test years (2002, 2013, 2021) is displayed.

**Figure 7:**
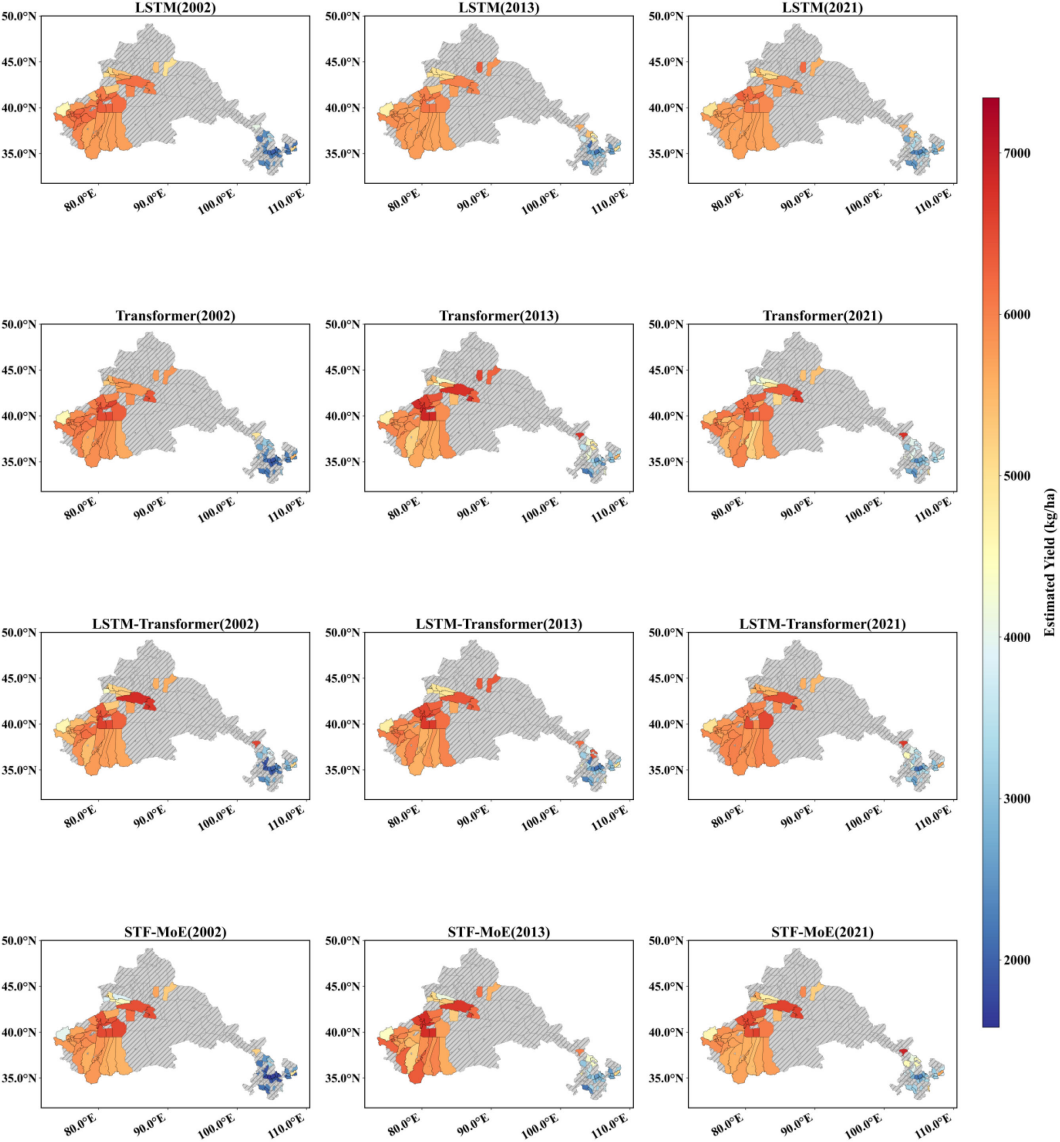
County-level yield estimation map. Red indicates high yields, and blue indicates low yields. The spatial distribution of estimations from four models across three independent test years (2002, 2013, 2021) is displayed.

The STF-MoE model estimated the spatial distribution of wheat yields in the study area for 2002, 2013, and 2021 (**Figures 6**, **7**) and was compared with baseline models. Results demonstrate that the STF-MoE superiorly captures spatial heterogeneity characteristics of wheat yields: **Figure 6** (prefecture-level) clearly distinguishes high-yield regions in the east/southeast from low-yield regions in the west/northwest, while **Figure 7** (county-level) reveals similar regional disparities. Compared to baseline models, the STF-MoE provides clearer delineation of yield classes and spatial transitions, with richer detail. Temporally, the STF-MoE also reflects interannual yield variability. Prefecture-level yield maps (**Figure 6**) visually illustrate interannual fluctuations in high-yield extent/intensity in core production zones (e.g., eastern regions) and growth trends in certain areas. For example, **Table 3** data indicate a significant yield increase in Zhoukou City, Henan Province from 2002 to 2021, which the STF-MoE largely captured, achieving an accuracy, where single sample accuracy is calculated as min(true value, predicted value)/max(true value, predicted value), exceeding 89% across all three years. At the county level (**Figure 7**), Zepu County, Xinjiang exhibited high yields in 2002 and 2013, with model accuracies reaching 90.6% and 97.5%, respectively; even in 2021, when actual yields declined, model accuracy remained at 94.3%. Collectively, the spatiotemporal estimation results of the STF-MoE model for the three independent test years (2002, 2013, 2021) exhibit good consistency with official statistical data from representative counties in **Table 3**. For instance, Bozhou City, Anhui Province and Dezhou City, Shandong Province maintained high estimation accuracies across all three years (Bozhou: 92.7%–99.7%; Dezhou: >93%). This confirms that the STF-MoE not only accurately identifies overall spatial patterns and distinguishes high-/low-yield regions but also reliably estimates specific yield levels across diverse regions and years. These results hold significant reference value for evaluating regional wheat production dynamics.

**Table 3:** Estimation accuracy of the STF-MoE model for selected counties/districts across three independent test years (AY, actual yield (kg/ha); EY, estimated yield).

Year	City	AY	EY	Accuracy
2002	Zepu (Xinjiang)	6361.0	5764.208	90.6%
	Lingtai (Gansu)	2434.0	2696.8855	90.3%
	Weinan (Shaanxi)	3184.0	4174.843	76.3%
	Zhoukou (Henan)	5852.371	5224.6763	89.3%
	Bozhou (Anhui)	4569.0	4721.2676	96.8%
	Deizhou (Shandong)	5639.0	6025.6777	93.6%
2013	Zepu (Xinjiang)	6180.0	6027.9014	97.5%
	Lingtai (Gansu)	2564.0	2729.755	93.9%
	Weinan (Shaanxi)	3307.0	3999.7224	82.7%
	Zhoukou (Henan)	7438.536	6772.1553	91.0%
	Bozhou (Anhui)	7572.0	7019.9785	92.7%
	Deizhou (Shandong)	7181.0	6912.157	96.3%
2021	Zepu (Xinjiang)	5401.0	5725.526	94.3%
	Lingtai (Gansu)	3425.0	3175.9827	92.7%
	Weinan (Shaanxi)	4401.803	4360.7534	99.1%
	Zhoukou (Henan)	7542.0	7308.282	96.9%
	Bozhou (Anhui)	7260.0	7280.166	99.7%
	Deizhou (Shandong)	7002.3335	7191.1284	97.4%

### 3.4 Early estimation capability of wheat yield

**Figure 8:**
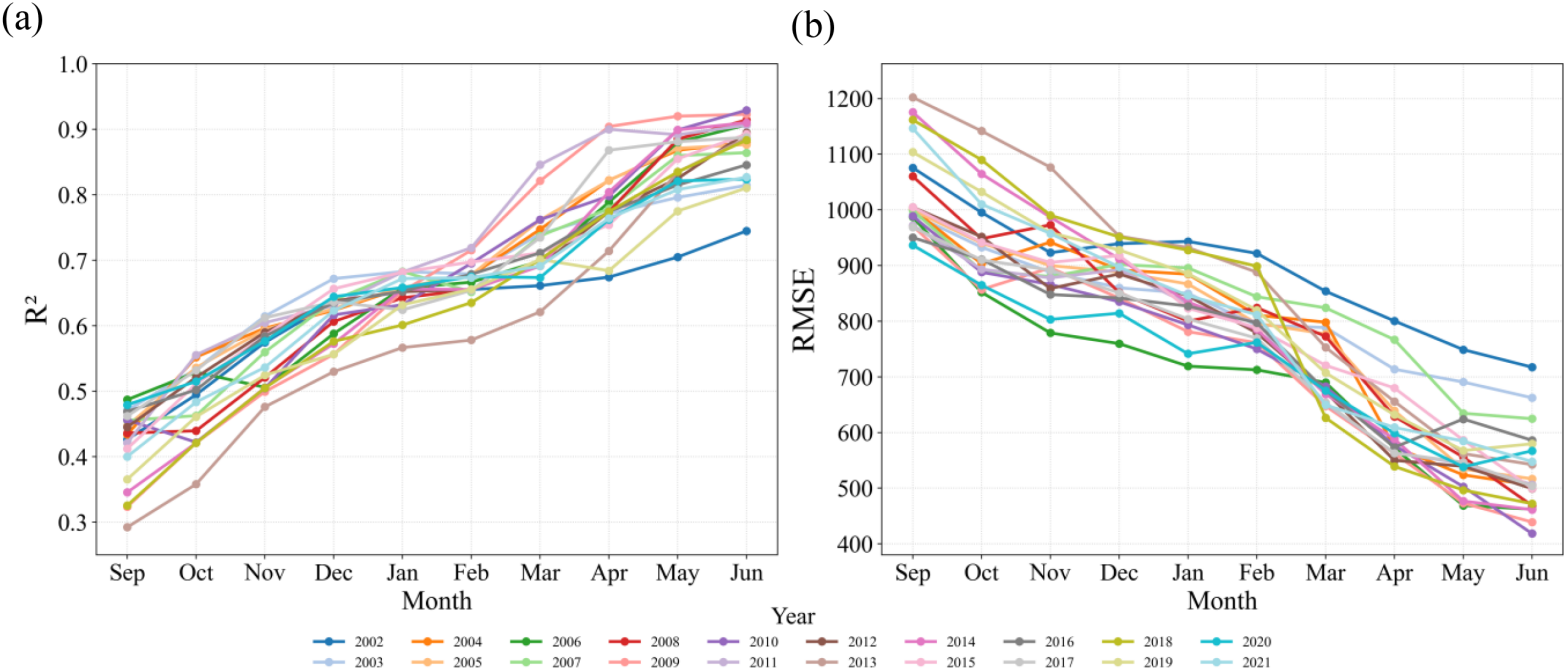
Verify test **(a)** R2 and **(b)** RMSE from wheat by month stage from 2002 to 2021.

**Figure 8** reveals the temporal evolution of R² and RMSE for the STF-MoE model in estimating seasonal wheat yield (September to June of the following year), demonstrating accuracy improvements driven by cumulative data: R² increased progressively, while RMSE decreased correspondingly. During the early growth phase (September–December), model R² ranged between 0.3–0.6, with RMSE generally exceeding 800 kg/ha. From the overwintering period to February, wheat growth slowed, resulting in limited accuracy gains; however, beginning in March—coinciding with rapid growth stages (regreening, jointing, and heading)—model performance improved markedly. Notably, in some years (e.g., 2009, 2011, 2017, 2021), R² reached high levels as early as April, highlighting early estimation potential. More commonly, as additional growth data accumulated, most years exhibited significant accuracy improvements by May, with R² rising sharply (e.g., from ≈0.7 in March to >0.8 in May for 2017) and RMSE declining substantially (typically from 700–900 kg/ha in March to<500–700 kg/ha in May). By June (harvest), R² consistently exceeded 0.8, and RMSE reached minimal values (mostly<600 kg/ha, some<500 kg/ha). This pronounced improvement in R² and RMSE from March to June clearly reflects significant physiological changes in wheat during critical growth stages: chlorophyll content, canopy structure, and photosynthetic intensity increase dynamically, while water demand peaks (Zhang et al., 2024; Liu et al., 2022). The indices selected in this study—NIRv (near-infrared reflectance vegetation index), Fpar (fraction of photosynthetically active radiation absorption), and RHum (relative humidity)—precisely capture these dynamics: NIRv and Fpar are highly sensitive to vegetation green biomass, chlorophyll status, and light-use efficiency, enabling accurate detection of canopy growth and photosynthetic enhancement, while RHum reflects atmospheric moisture stress during this critical period. The tight coupling between these remote sensing features and wheat’s phenological/ecological processes provides the model with dynamic information closely linked to final yield formation, thereby enabling substantial accuracy gains during these stages. These results confirm the STF-MoE model’s capability to leverage cumulative data for reliable yield estimation 1–2 months prior to harvest.

### 3.5 Importance of individual indicators in yield estimation

**Figure 9:**
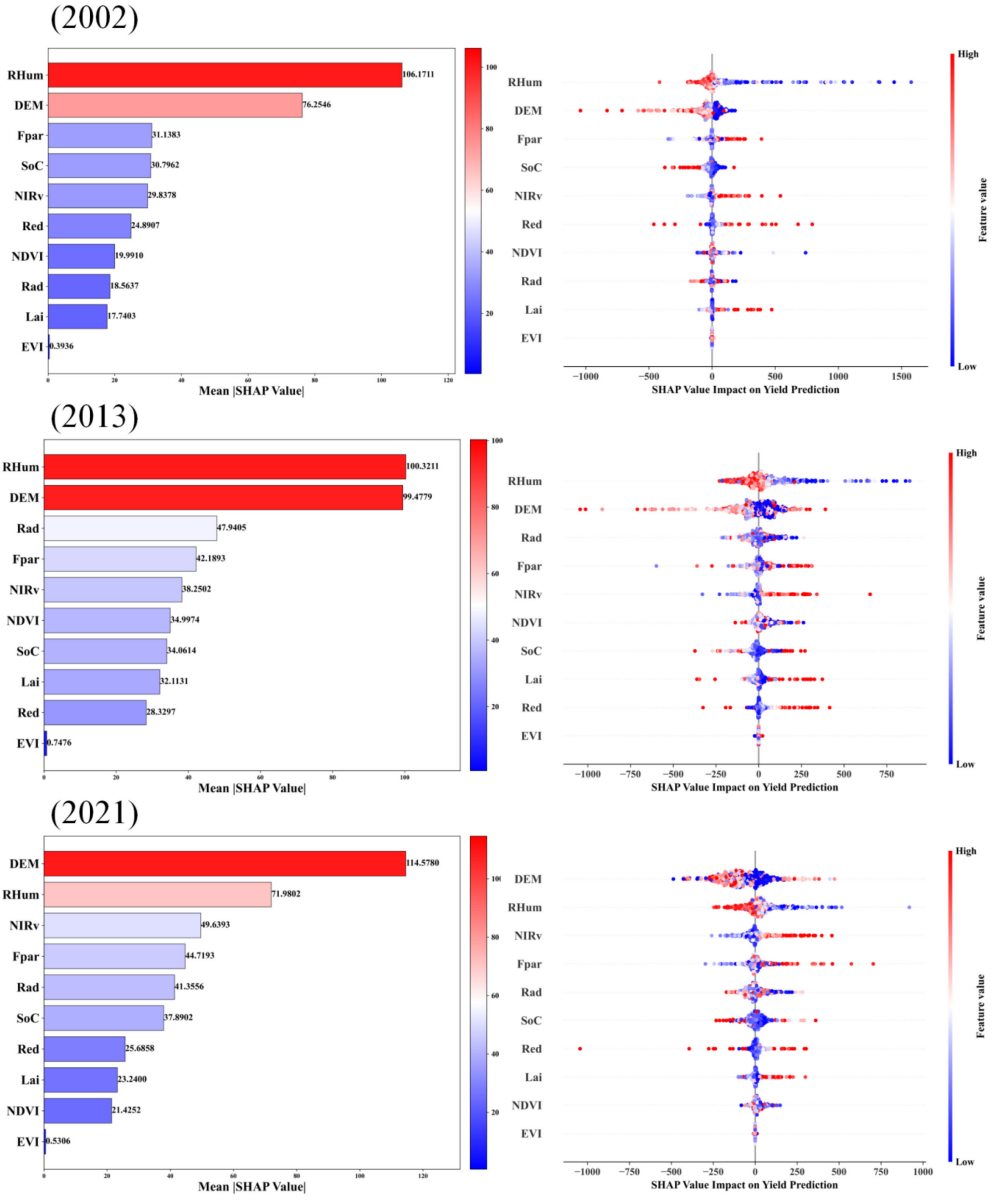
Average SHAP values of features across three independent test years and SHAP value impacts of features on wheat yield estimation.

**Figure 9** presents the SHAP analysis results, revealing the contributions of input features to the STF-MoE model’s wheat yield estimation across three test years (2002, 2013, 2021). RHum (relative humidity) and DEM (digital elevation model) consistently emerged as the top two drivers with the highest mean SHAP values. The SHAP dependence plots (right panels of **Figure 9**) demonstrate that higher RHum values (red points) stably corresponded to negative SHAP values in all years, indicating that elevated relative humidity consistently exerted a negative impact on yield estimations. This aligns with preliminary analyses in Section 3.1 (Exploratory Feature Data Analysis) and may be attributed to disease risks, pollination interference, or adverse effects on crop maturation and drying under high humidity. DEM, reflecting macro-topographic influences, exhibited interannual variability in its impact patterns. For instance, in 2002, higher DEM values (red points) primarily corresponded to negative SHAP values, while in 2013 and 2021, higher DEM values shifted to positive SHAP associations, suggesting that elevated areas in these years were linked to higher yield estimations due to integrated environmental advantages (e.g., improved drainage, sunlight exposure, or reduced pest/disease risks). Remote sensing indices closely tied to vegetation growth and photosynthetic capacity—Fpar (fraction of photosynthetically active radiation absorption) and NIRv (near-infrared reflectance vegetation index)—also displayed high SHAP values. Higher Fpar and NIRv values (red points) generally aligned with positive SHAP values, signifying superior vegetation status and photosynthetic potential that positively influenced yield (Murchie et al., 2009). Similarly, elevated Rad (photosynthetically active radiation) and SoC (soil organic carbon) values (red points) predominantly contributed positively to yield, as reflected in their positive SHAP values. Notably, while the relative importance of key drivers remained broadly consistent across years, their specific SHAP magnitudes and rankings varied interannually. The directional shift in DEM’s influence (negative in 2002 vs. positive in 2013/2021) exemplifies the complexity of feature impacts. Additionally, even for features with consistent directional effects like RHum, the intensity of their negative impacts (absolute negative SHAP values) varied across samples. Lower RHum values (blue points) mostly aligned with positive SHAP values, though instances of negligible or slightly negative effects were observed, reflecting potential nonlinear responses or complex feature interactions under specific conditions.

## 4 Discussion

### 4.1 Error discussion of the model in wheat yield estimation

**Figure 10:**
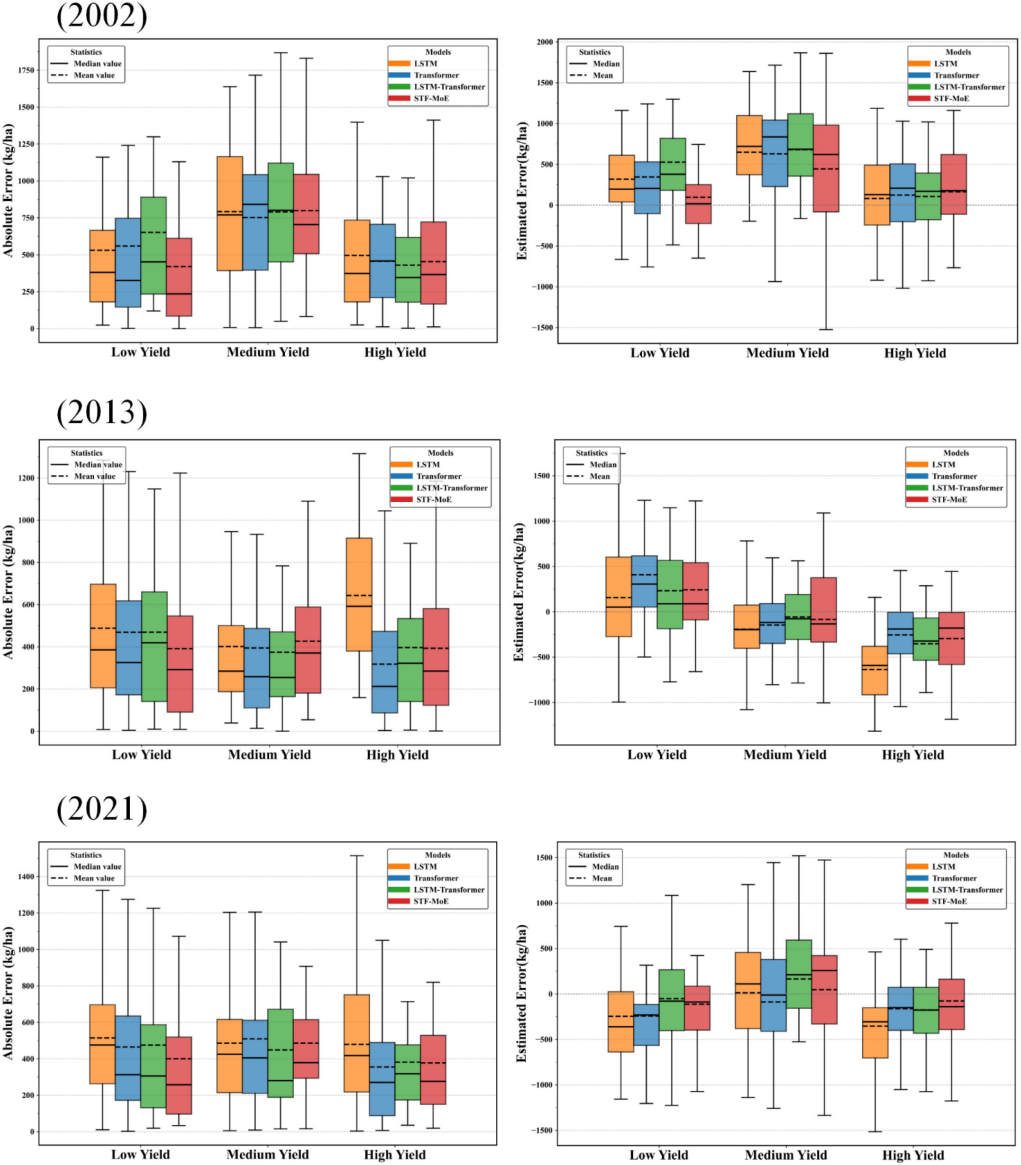
Comparison of absolute and raw errors across different yield ranges in three independent test years (left panel: boxplots of absolute errors; right panel: boxplots of raw errors).

A comprehensive analysis of the error characteristics of the STF-MoE model in wheat yield estimation reveals its overall performance advantages while highlighting notable error patterns. Firstly, in terms of macro-level metrics, the STF-MoE achieved a mean R² of 0.8712 and a mean RMSE of 529.0713 kg/ha during year-by-year rolling tests from 2002 to 2021 (**Supplementary Tables S1**,**S2**), outperforming baseline models such as LSTM and Transformer and demonstrating competitive average predictive accuracy over long-term sequences. In critical independent year tests (**Table 2**), such as 2013 (an extreme weather year), the STF-MoE exhibited robustness with an R² of 0.887 and an RMSE of 542.4 kg/ha. Error boxplots in **Figure 10** further corroborate these findings at a granular level: in 2013, the STF-MoE’s median raw errors approached zero across low-, medium-, and high-yield intervals, with tightly distributed absolute errors, indicating effective control of estimation biases across yield levels under complex conditions. County-level data in **Table 3** validate the model’s potential, such as 99.7% accuracy in Bozhou, Anhui, and 97.4% accuracy in Dezhou, Shandong in 2021, highlighting its high-precision estimation capability in specific regions.

However, a deeper dissection of error sources and distributions reveals that the STF-MoE’s estimation errors do not consistently achieve optimal performance. Scatter plots in **Figure 5** visually demonstrate varying degrees of dispersion between predicted and actual values, particularly at yield extremes (low-yield and high-yield regions), with notable deviations from the ideal fit line. Error boxplots in **Figure 10** quantify this: in high-yield regions for 2013 and 2021, the STF-MoE’s median raw errors were negative, unambiguously revealing a systematic underestimation tendency, while larger absolute error ranges reflect heightened uncertainty in these areas. County-level accuracy data in **Table 2** echo this observation, such as underestimation in Zhoukou, Henan (2013), where actual yields were relatively high. Furthermore, interannual performance variability remains a critical concern, as shown in (**Supplementary Tables S1** and **S2**): R² fluctuated by ≈0.18 and RMSE by nearly 280 kg/ha across years, indicating room for improvement in model stability.

Collectively, the STF-MoE model has made significant strides in wheat yield estimation error control, particularly commendable in its average performance and resilience under specific complex conditions. Nevertheless, its error profile exposes challenges in handling yield extremes (especially underestimation in high-yield regions) and maintaining consistency across years and regions. Specifically, the model exhibited relatively larger errors in certain low-yield areas (e.g., Weinan, Shaanxi in 2002) and high-yield samples (e.g., select high-yield cases in 2002 and 2021), alongside persistent interannual performance fluctuations.

### 4.2 Ablation analysis discussion for the STF-MoE model

To systematically evaluate the contributions of key architectural components within the STF-MoE model, specifically the Mixture of Experts (MoE) architecture and its sparse gating mechanism, an ablation study was conducted. The results, detailed in **Table 4** and derived from the 2021 test set, form the basis of this discussion. FLOPs (Floating Point Operations) are employed throughout this analysis as a metric for computational complexity, where lower values generally signify greater operational efficiency. The full STF-MoE model (FLOPs: 32.59M) was compared to an “STF (without MoE)” variant (FLOPs: 31.98M). Superior performance in RMSE (547.7 vs. 578.4 kg·ha^-^¹), R² (0.827 vs. 0.807), and MAE (420.3 vs. 463.9 kg·ha^-^¹) was observed for the STF-MoE, despite a marginal FLOPs increase, initially suggesting the MoE’s efficacy in enhancing predictive accuracy. To further probe the MoE fusion mechanism, an “STF-SingleExpert” configuration (FLOPs: 32.38M), utilizing a single general-purpose expert (detailed in Section 2.3.3 (1)), was examined. While this single-expert model outperformed the STF model lacking any MoE structure, it was notably surpassed by the complete STF-MoE model. This critically indicates that the MoE’s strength lies in its dynamic selection and fusion of multiple heterogeneous experts, allowing for more flexible and superior feature processing than a single expert, thereby substantiating the MoE fusion approach’s effectiveness. The impact of the sparse gating mechanism (Top-2 expert selection) was assessed by comparing the full STF-MoE with the “STF-MoE (without Sparse Gating, Top-2 removed)” variant. The performance metrics (RMSE, R², and MAE) indicated comparable predictive accuracy between these two configurations (e.g., R² of 0.827 for STF-MoE vs. 0.822 for the variant without sparse gating). However, a notable reduction in computational complexity was observed when sparse gating was employed, as evidenced by the lower FLOPs for the STF-MoE model (32.59M) compared to the model without sparse gating (33.51M). This finding underscores the primary advantage and utility of the sparse gating mechanism: it effectively curtails the model’s computational demands while maintaining a high level of predictive performance.

**Table 4:** Ablation analysis conducted on the 2021 test set.

Experimental combinations	RMSE (kg·ha^–1^)	R^2^	MAE (kg·ha^–1^)	FLOPs
STF-MoE	547.7	0.827	420.3	32.59M
STF (without MoE)	578.4	0.807	463.9	31.98 M
STF-SingleExpert (MoE removed, weighted by a single expert)	568.9	0.8130	430.0	32.38M
STF-MoE (without Sparse Gating, Top-2 removed)	554.9	0.822	416.9	33.51M

### 4.3 Future research and improvements

While the STF-MoE model demonstrates strong performance in wheat yield estimation, it faces challenges arising from objective factors and offers avenues for future refinement. Firstly, to address the model’s underestimation in high-yield regions, larger errors in low-yield areas, and interannual performance variability, future efforts should prioritize enhancing its adaptability to extreme yield values and spatiotemporal heterogeneity. This may involve integrating high spatiotemporal-resolution data sources that better capture localized microclimates, extreme weather events, sudden pest/disease outbreaks, and fine-scale agricultural practices (e.g., irrigation/fertilization variations), alongside developing more effective feature fusion and representation methods. Secondly, given the practical demands of large-scale regional applications, computational cost and inference speed are critical considerations. Although the STF-MoE optimizes efficiency via Top-K sparse gating mechanisms, its complex architecture—incorporating Transformers and multiple expert networks—may impose computational overhead. Future research could explore model compression techniques (e.g., knowledge distillation, parameter pruning), lightweight network designs (e.g., efficient attention mechanisms or expert networks), or hardware acceleration (e.g., GPU/TPU optimization) to substantially reduce training/inference time, facilitating operational deployment and rapid response. Additionally, continuously expanding ground-truth yield datasets and high-precision remote sensing data across broader geographical regions and longer time series will be crucial to validate and enhance the model’s generalizability and robustness. This remains a pivotal direction for future research.

## 5 Conclusions

The dynamic gated deep learning model (STF-MoE), developed in this study through the integration of multi-source remote sensing data and a heterogeneous mixture-of-experts (MoE) mechanism built upon an LSTM-Transformer framework, significantly enhanced the accuracy and robustness of yield estimation in six major wheat-producing provinces in China. Year-by-year rolling experiments from 2002 to 2021 demonstrated that the STF-MoE outperformed baseline models in key metrics, achieving a mean R² of 0.8712 and a mean RMSE of 529.0713 kg/ha, while effectively capturing spatiotemporal yield heterogeneity and controlling errors across yield levels, particularly exhibiting strong adaptability during extreme weather years (e.g., 2013). SHAP-based interpretability analysis revealed the critical driving roles of environmental factors such as relative humidity (RHum) and digital elevation model (DEM) in yield estimation. Despite remaining challenges in handling yield extremes and maintaining interannual stability, alongside the need for further computational efficiency optimization to enable large-scale deployment, the STF-MoE model provides a valuable deep learning solution for precise crop yield estimation in complex agricultural ecosystems, offering robust technical support for food security assurance and agricultural management optimization.

## Data Availability

The original contributions presented in the study are included in the article/**Supplementary Material**. Further inquiries can be directed to the corresponding authors.
