# Supplementary material for: Dynamic gating-enhanced deep learning model with multi-source remote sensing synergy for optimizing wheat yield estimation
Source: Front Plant Sci. 2025 Jul 21;16:1640806. doi: 10.3389/fpls.2025.1640806 (PMC12318938; doi:10.3389/fpls.2025.1640806)
Supplement: Supplementary file 1 [file Table1.docx]

Supplementary Material

**S1.** Test R^2^ for STF-MoE, Transformer, LSTM and LSTM-Transformer models between 2002 and 2021.

| **Year** | **Transformer** | **LSTM** | **LSTM-Transformer** | **STF-MoE** |
| --- | --- | --- | --- | --- |
| 2002 | 0.7131 | 0.7100 | 0.6982 | 0.7449 |
| 2003 | 0.7739 | 0.8045 | 0.7648 | 0.8146 |
| 2004 | 0.8663 | 0.8537 | 0.8767 | 0.8800 |
| 2005 | 0.8838 | 0.8815 | 0.8730 | 0.8769 |
| 2006 | 0.8751 | 0.8962 | 0.8942 | 0.9069 |
| 2007 | 0.8554 | 0.8504 | 0.8370 | 0.8644 |
| 2008 | 0.8859 | 0.8563 | 0.8852 | 0.9144 |
| 2009 | 0.8898 | 0.8844 | 0.9090 | 0.9230 |
| 2010 | 0.8752 | 0.8686 | 0.9199 | 0.9293 |
| 2011 | 0.8714 | 0.8729 | 0.8943 | 0.9064 |
| 2012 | 0.8818 | 0.8542 | 0.8792 | 0.8950 |
| 2013 | 0.8776 | 0.8421 | 0.8735 | 0.8866 |
| 2014 | 0.8619 | 0.8573 | 0.8856 | 0.9103 |
| 2015 | 0.9069 | 0.8739 | 0.8847 | 0.8927 |
| 2016 | 0.8931 | 0.8947 | 0.8992 | 0.8458 |
| 2017 | 0.8982 | 0.8626 | 0.8843 | 0.8878 |
| 2018 | 0.8856 | 0.8812 | 0.8831 | 0.8836 |
| 2019 | 0.8136 | 0.7770 | 0.7694 | 0.8105 |
| 2020 | 0.7841 | 0.7871 | 0.7801 | 0.8240 |
| 2021 | 0.7943 | 0.7883 | 0.8052 | 0.8270 |
| **Avg** | **0.8544** | **0.8448** | **0.8548** | **0.8712** |

**S2.** Test RMSE for STF-MoE, Transformer, LSTM and LSTM-Transformer models between 2002 and 2021.

| **Year** | **Transformer** | **LSTM** | **LSTM-Transformer** | **STF-MoE** |
| --- | --- | --- | --- | --- |
| 2002 | 760.7584 | 764.8146 | 780.2812 | 717.3959 |
| 2003 | 731.4404 | 680.2125 | 746.0911 | 662.4047 |
| 2004 | 533.5346 | 558.1954 | 512.3229 | 505.3699 |
| 2005 | 502.2914 | 507.2141 | 525.1215 | 516.8458 |
| 2006 | 466.5224 | 487.9232 | 492.5618 | 462.2039 |
| 2007 | 645.2794 | 656.3842 | 685.0852 | 624.7763 |
| 2008 | 541.4730 | 607.6418 | 543.1016 | 468.8405 |
| 2009 | 448.1072 | 462.9242 | 477.3924 | 439.1295 |
| 2010 | 430.4435 | 570.7213 | 445.6823 | 418.4544 |
| 2011 | 492.7029 | 590.1406 | 538.1351 | 506.4743 |
| 2012 | 482.6250 | 588.0107 | 535.3995 | 499.1626 |
| 2013 | 563.6121 | 640.0547 | 572.7662 | 542.4078 |
| 2014 | 457.1627 | 493.4602 | 520.9586 | 461.3179 |
| 2015 | 465.5592 | 541.8525 | 518.1771 | 499.7336 |
| 2016 | 487.7690 | 484.2502 | 473.6413 | 586.0394 |
| 2017 | 480.2482 | 557.9274 | 512.0264 | 504.1393 |
| 2018 | 467.9953 | 476.9109 | 473.2759 | 472.1801 |
| 2019 | 574.8759 | 628.7850 | 639.4075 | 579.7047 |
| 2020 | 622.3213 | 618.1231 | 626.9331 | 567.1251 |
| 2021 | 596.9421 | 606.2319 | 581.2121 | 547.7210 |
| **Avg** | **537.5832** | **586.1364** | **559.9786** | **529.0713** |
